# Electronic Alert Systems for Patients With Acute Kidney Injury: A Systematic Review and Meta-Analysis

**DOI:** 10.1001/jamanetworkopen.2024.30401

**Published:** 2024-08-27

**Authors:** Jia-Jin Chen, Tao-Han Lee, Ming-Jen Chan, Tsung-Yu Tsai, Pei-Chun Fan, Cheng-Chia Lee, Vin-Cent Wu, Yu-Kang Tu, Chih-Hsiang Chang

**Affiliations:** 1 Kidney Research Center, Department of Nephrology, Chang Gung Memorial Hospital, Linkou Branch, Taoyuan, Taiwan; 2 College of Medicine, Chang Gung University, Taoyuan, Taiwan; 3 Department of Nephrology, Chansn Hospital, Taoyuan City, Taiwan; 4 Department of Internal Medicine, National Taiwan University Hospital, Taipei, Taiwan; 5 National Taiwan University Study Group on Acute Renal Failure, Taipei, Taiwan; 6 Institute of Epidemiology and Preventive Medicine, College of Public Health, National Taiwan University

## Abstract

**Importance:**

The acute kidney injury (AKI) electronic alert (e-alert) system was hypothesized to improve the outcomes of AKI. However, its association with different patient outcomes and clinical practice patterns remains systematically unexplored.

**Objective:**

To assess the association of AKI e-alerts with patient outcomes (mortality, AKI progression, dialysis, and kidney recovery) and clinical practice patterns.

**Data Sources:**

A search of Embase and PubMed on March 18, 2024, and a search of the Cochrane Library on March 20, 2024, to identify all relevant studies. There were no limitations on language or article types.

**Study Selection:**

Studies evaluating the specified outcomes in adult patients with AKI comparing AKI e-alerts with standard care or no e-alerts were included. Studies were excluded if they were duplicate cohorts, had insufficient outcome data, or had no control group.

**Data Extraction and Synthesis:**

Two investigators independently extracted data and assessed bias. The systematic review and meta-analysis followed the PRISMA guidelines. Random-effects model meta-analysis, with predefined subgroup analysis and trial sequential analyses, were conducted.

**Main Outcomes and Measures:**

Primary outcomes included mortality, AKI progression, dialysis, and kidney recovery. Secondary outcomes were nephrologist consultations, post-AKI exposure to nonsteroidal anti-inflammatory drugs (NSAID), post-AKI angiotensin-converting enzyme inhibitor and/or angiotensin receptor blocker (ACEI/ARB) prescription, hospital length of stay, costs, and AKI documentation.

**Results:**

Thirteen unique studies with 41 837 unique patients were included (mean age range, 60.5-79.0 years]; 29.3%-48.5% female). The risk ratios (RRs) for the AKI e-alerts group compared with standard care were 0.96 for mortality (95% CI, 0.89-1.03), 0.91 for AKI stage progression (95% CI, 0.84-0.99), 1.16 for dialysis (95% CI, 1.05-1.28), and 1.13 for kidney recovery (95% CI, 0.86-1.49). The AKI e-alerts group had RRs of 1.45 (95% CI, 1.04-2.02) for nephrologist consultation, 0.75 (95% CI, 0.59-0.95) for post-AKI NSAID exposure. The pooled RR for post-AKI ACEI/ARB exposure in the AKI e-alerts group compared with the control group was 0.91 (95% CI, 0.78-1.06) and 1.28 (95% CI, 1.04-1.58) for AKI documentation. Use of AKI e-alerts was not associated with lower hospital length of stay (mean difference, −0.09 [95% CI, −0.47 to 0.30] days) or lower cost (mean difference, US $655.26 [95% CI, −$656.98 to $1967.5]) but was associated with greater AKI documentation (RR, 1.28 [95% CI, 1.04-1.58]). Trial sequential analysis confirmed true-positive results of AKI e-alerts on increased nephrologist consultations and reduced post-AKI NSAID exposure and its lack of association with mortality.

**Conclusions and Relevance:**

In this systematic review and meta-analysis, AKI e-alerts were not associated with a lower risk for mortality but were associated with changes in clinical practices. They were associated with lower risk for AKI progression. Further research is needed to confirm these results and integrate early AKI markers or prediction models to improve outcomes.

## Key Points

**Question:**

Are electronic alerts (e-alerts) for acute kidney injury (AKI) in the electronic health record associated with patient outcomes or clinical practice patterns?

**Findings:**

In this systematic review and meta-analysis of 13 unique studies with 41 837 unique patients, AKI e-alerts were not associated with a lower risk for mortality but were associated with a lower risk for AKI progression compared with standard care. There were associations between e-alerts and clinical practice patterns, including increased nephrologist consultations, dialysis, and AKI documentation and decreased post-AKI exposure to nonsteroidal anti-inflammatory drugs.

**Meaning:**

These findings suggest that AKI e-alerts are associated with changes to clinical practice patterns and lower risk for AKI progression, although more research is needed to support this conclusion.

## Teaser

This meta-analysis assesses the association between electronic alerts and patient survival, kidney outcomes, clinical practice patterns, and associated outcomes such as medical costs and hospital length of stay in patients with acute kidney injury.

## Introduction

Acute kidney injury (AKI) is a common complication in hospitalized patients, leading to increased comorbidities, health care costs, and both short- and long-term mortality.^1,2,3^ The introduction of electronic health record systems has enabled early detection of AKI through electronic alerts (e-alerts), considered potential interventions to reduce AKI-related complications and improve outcomes. Consequently, the AKI e-alert system was initially launched in the US and the United Kingdom, later expanding globally.^4,5,6,7^

A 2012 study by Colpaert et al^8^ using RIFLE (risk, injury, failure, loss of kidney function, and end-stage kidney disease) criteria showed that AKI e-alerts could enhance short-term renal outcomes and timely interventions. The 27th Acute Disease Quality Initiative consensus also highlighted that “AKI alerts driven by concrete criteria improve early detection and prompt AKI management.”^9^ Nevertheless, a 2017 published meta-analysis^6^ and subsequent randomized clinical trials (RCTs) and non-RCTs, including Electronic Alerting for Acute Kidney Injury Amelioration (ELAIA)–1^10^ and ELAIA-2,^11^ questioned their impact on mortality. Despite assumptions about their efficacy in improving AKI outcomes and care, it remains uncertain whether AKI e-alerts, alone or with care bundles, are associated with lower mortality, AKI severity, or the need for kidney replacement or whether they impact clinical practices.

Given the lack of systematic analysis for several associated outcomes, an updated meta-analysis including recently published studies^10,11,12^ is warranted. In the present study, we performed a systematic review and meta-analysis, incorporating subgroup analysis and trial sequential analysis using evidence-based medicine methods to assess the association between AKI e-alerts and patient survival, kidney outcomes, clinical practice patterns, and associated outcomes such as medical costs and hospital length of stay (LOS).

## Methods

### Literature Search Strategy

This systematic review and meta-analysis was performed in accordance with the Preferred Reporting Items for Systematic Reviews and Meta-Analyses (PRISMA) statement and checklist. We registered the protocol in PROSPERO (CRD42024527189). Two investigators (J.-J.C. and T.-H.L.) systematically and independently conducted a review of published data on outcomes in patients with AKI e-alerts. A search of PubMed and Embase was performed on March 18, 2024, and the Cochrane Library was searched on March 20, 2024, to identify all relevant studies. Detailed search strategies, including search terms specific to each source, are provided in eTable 1 in Supplement 1. There were no limitations on language or article types.

### Study Eligibility Criteria

After removing duplicates, titles and abstracts were screened by 2 reviewers (J.-J.C. and T.-H.L.) for relevance. Full texts of potentially relevant articles were then reviewed for eligibility. Inclusion criteria required studies to involve adults, compare AKI e-alert groups with non–e-alert groups, and report on any of the primary or secondary outcomes. For eligibility disagreements, a third reviewer (C.-H.C.) was consulted for consensus. Exclusions were made for duplicate cohorts, insufficient outcome data, or absence of a control group.

### Data Extraction and Outcome Measurement

The 2 investigators (J.-J.C. and T.-H.L.) independently extracted data (author[s], publication year, design, location, AKI care bundle presence, sample size, AKI criteria, mean age, proportion of population that was female) and outcomes from each study. For binary outcomes, participant and event numbers were noted; for continuous outcomes, mean and SD were extracted or calculated from median (IQR). Discrepancies were resolved through discussion with a third investigator (P.-C.F.).

### Outcomes

This systematic review and meta-analysis evaluated the differences between AKI e-alerts vs standard care or no e-alerts for patient outcomes or clinical practice patterns. Primary outcomes included mortality and dialysis after AKI (prioritizing 28-day or 30-day, then 60-day, 90-day, and in-hospital mortality and dialysis), AKI stage progression, and kidney recovery after AKI. Secondary outcomes were nephrologist consultations, post-AKI exposure to nonsteroidal anti-inflammatory drugs (NSAIDs), post-AKI angiotensin-converting enzyme inhibitor and/or angiotensin receptor blocker (ACEI/ARB) prescription, AKI documentation, post-AKI intravenous fluid prescription, hospital LOS, and medical costs.

### Statistical Analysis

In the R meta package, the metabin and metacont functions were used for binary and continuous outcomes, respectively.^13^ We applied a random-effects model using the inverse variance method. Between-study variance was estimated using the restricted maximum-likelihood estimator method, while the DerSimonian and Laird method estimated the 95% CI of the effect. We assessed the overall effect using pooled risk ratios (RRs) for binary outcomes and mean differences for continuous outcomes. Heterogeneity was evaluated with the *I*^2^ statistic. Small study bias was examined using funnel plots and the Egger test via the metabias function.^14^ Analyses were conducted in R, version 4.2.2 (R Program for Statistical Computing [October 31, 2022]), with 2-sided *P* < .05 considered statistically significant.

#### Prespecified Subgroup Analysis

In our analysis, we differentiated studies as RCTs vs non-RCTs. We hypothesized that AKI e-alerts, combined with care recommendations or bundles, might be associated with patient outcomes. To explore this, we performed a subgroup analysis, dividing studies into those using e-alerts with AKI care bundles or recommendations and those using e-alerts alone. For studies reporting mortality outcomes over different time periods, we additionally conducted a subgroup analysis based on the specific time period.

#### Trial Sequential Analysis and Sensitivity Analysis

To determine whether the primary outcome conclusions of our meta-analysis were premature, we performed trial sequential analysis (TSA) using TSA software, version 0.9.5.10 beta.^15^ A more detailed description is found in eAppendix 1 in Supplement 1.

Considering that the traditional DerSimonian and Laird method might underestimate between-study heterogeneity and the relatively small number of enrolled studies, we performed sensitivity analyses for binary outcomes using the Hartung-Knapp method and beta-binomial bayesian meta-analysis. The beta-binomial bayesian meta-analysis was conducted using R software and the JAGS (Just Another Gibbs Sampler) program, version 4.3.2 (GNU General Public License). Additionally, we conducted further TSA including only RCTs for both primary and secondary outcomes that showed associations in the conventional meta-analysis.

#### Risk of Bias and Certainty of Evidence Assessment and Confidence

We assessed the risk of bias using RoB 2.0 (a revised tool to assess risk of bias in randomized trials)^16^ and ROBINS-I tool (Risk of Bias in Nonrandomized Studies of Interventions)^17^ for included RCTs and non-RCTs, respectively. Two independent reviewers (J.-J.C. and T.-H.L.) assessed the bias according to each domain, and the disagreements between the reviewers were resolved by discussion with another author (P.-C.F.). The quality of evidence was evaluated based on the guidelines of the GRADE (Grades of Recommendation, Assessment, Development, and Evaluation) Working Group.^18,19^

## Results

### Search Results and Study Characteristics

A flowchart of the literature search is provided in eFigure 1 in Supplement 1). The electronic database search identified 189 potentially eligible studies from PubMed, 98 from Embase, and 42 from the Cochrane Library. After removing duplicate articles, the remaining 259 articles were screened. After screening the titles and abstracts, the full texts of 34 studies were reviewed to assess their eligibility. After excluding studies for various reasons (eTable 2 in Supplement 1), 13 unique studies including 41 837 unique patients^7,8,10,11,12,20,21,22,23,24,25,26,27^ were included for analysis.

Table 1 summarizes the characteristics of the included studies. Patients’ mean ages varied from 60.5 to 79.0 years, with female representation between 29.3% and 48.5% and male representation between 51.5% and 70.7%. Among the 13 studies, 6 were RCTs,^10,11,12,23,26,27^ 4 were prospective cohort studies,^7,8,22,25^ and 3 were retrospective cohort studies.^20,21,24^ All but 1 study^8^ adhered to Kidney Disease: Improving Global Outcomes (KDIGO) criteria for AKI, with the exception using RIFLE criteria. Additionally, 8 studies^7,11,12,20,21,22,23,25^ provided AKI management recommendations or care bundles alongside AKI e-alerts. Further details on inclusion and exclusion criteria and AKI care recommendations are available in eTable 3 in Supplement 1.

**Table 1.** Baseline Characteristics of Included Studies

Source	Study years	Design	Country	Type of location	Participant mean (SD) age, y^a^	Female, %	Baseline Cr level, mg/dL	AKI bundle care or suggestion	AKI criteria (definition)	AKI progression	Total No. of participants	Mortality period^b^
Atia et al,^20^ 2023	2013 to 2017	Retrospective cohort with propensity score match	United Kingdom	Single-center	NR	47.8	NR	Included	KDIGO (Cr level)	Progression to higher stage in 7 d	15 566	In hospital
Assem et al,^21^ 2023	2018 and 2020	Retrospective cohort	Germany	Single-center	NR	NR	NR	Included	KDIGO (Cr level)	NA	243	In hospital
Colpaert et al,^8^ 2012	2007 to 2007	Prospective cohort	Belgium	Single-center	61.4	38.4	0.82	Not included	RIFLE (Cr level and UOP)	NA	1079	28 d
Hodgson et al,^22^ 2018	2014 to 2016	Prospective cohort	United Kingdom	Multicenter	NR	NR	NR	Included	KDIGO (Cr level)	NA	1177	In hospital
Iwers et al,^23^ 2023	2019 to 2022	Cluster RCT	Germany	Single-center	79	46	1.3	Included	KDIGO (Cr level)	NA	200	In hospital
Kotwal et al,^7^ 2023	2019 to 2019	Prospective cohort	Australian	Multicenter	74 (17)	46	1	Included	KDIGO (Cr level)	NA	639	In hospital
Li et al,^12^ 2024	2019 to 2021	Double-blind RCT	China	Single-center	64.5	29.3	0.87	Included	KDIGO (Cr level)	In hospital	2208	28 d
Park et al,^24^ 2018	2013 to 2015	Retrospective cohort	Korea	Single-center	65.6	47.5	0.67	Not included	KDIGO (Cr level)	NA	3193	NA
Tome et al,^25^ 2022	2018 to 2018	Prospective cohort	Brazil	Single-center	66.0 (16.3)	42.1	1.1	Included	KDIGO (Cr level)	Progression to higher stage	3174	30 d
Wilson et al,^26^ 2015	2013 to 2014	Single-blind RCT	US	Single-center	60.5 (16.5)^c^	44.6	0.9	Not included	KDIGO (Cr level)	NA	2393	30 d
Wilson et al,^10^ 2021	2018 to 2019	Double-blind RCT	US	Multicenter	71.2	47.8	1.5	Not included	KDIGO (Cr level)	Progression to higher stage in hospital	6030	14 d
Wilson et al,^11^ 2023	2016 to 2021	Open-label RCT	US	Multicenter	70	48.5	1.2	Included	KDIGO (Cr level)	Progression to higher stage in hospital	5060	In hospital
Wu et al,^27^ 2018	2016 to 2016	Double-blind RCT	China	Single-center	62.9	35.1	0.9	Not included	KDIGO (Cr level)	NA	875	In hospital

Abbreviations: AKI, acute kidney injury; Cr, creatinine; KDIGO, Kidney Disease: Improving Global Outcomes; NA, nonapplicable; NR, not reported; RCT, randomized clinical trial; RIFLE, risk, injury, failure, loss of kidney function, and end-stage kidney disease; UOP, urine output.

SI conversion factor: To convert creatinine to μmol/L, multiply by 88.4.

^a^ Most studies did not provide overall SD of the mean age of enrolled participants; RCTs might only provide the IQR of each group. Some provide overall median age and IQR; therefore, the SD was calculated from the IQR.

^b^ If a study reported mortality outcomes for different time periods, they were prioritized in the following order: 28 days or 30 days, then 60 days, 90 days, and finally in-hospital mortality and/or dialysis.

^c^ Calculated from the mean and SD of 2 groups.

### Quality of Included Studies

The RoB 2.0 and ROBINS-I assessments indicated varied risk of bias across the studies, with 8 of 13 studies presenting low to moderate risk. For RCTs, overall quality was ranked as low risk for 4 studies (66.7%),^10,11,12,26^ of some concern for 1 study (16.7%),^27^ and of high concern for 1 study (16.7%)^23^ (eFigure 2 in Supplement 1). For non-RCTs, the overall quality was low risk for 1 study (14.3%),^20^ of moderate concern for 3 studies (42.9%),^7,8,25^ and of high concern for 3 studies (42.9%)^21,22,24^ (eFigure 3 in Supplement 1) (see details in eAppendix 2 in Supplement 1).

### Primary Outcomes: Mortality, AKI Stage Progression, Dialysis, and Kidney Recovery

**Figure 1.**
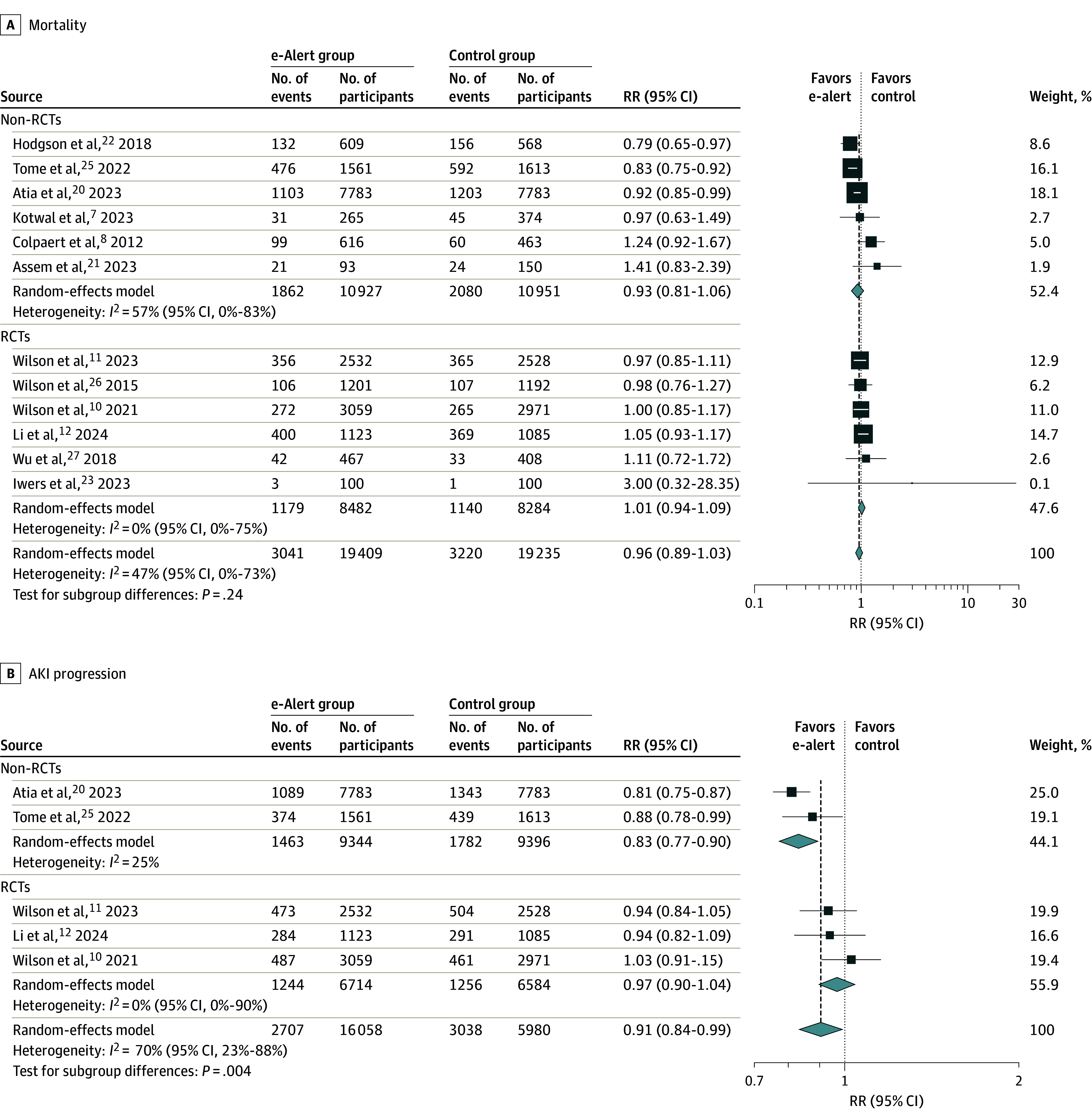
Association of Acute Kidney Injury (AKI) Electronic Alerts (e-Alerts) With Mortality and AKI Progression RCT indicates randomized clinical trial; RR, risk ratio. Diamonds indicate heterogeneity; different marker sizes, weights.

In 12 studies with 38 644 participants,^7,8,10,11,12,20,21,22,23,25,26,27^ the pooled mortality rate in the e-alert group was 15.7% (3041 of 19 409) and the pooled mortality rate in the control group was 16.7% (3220 of 19 235). Use of AKI e-alerts was not associated with a significant difference in mortality compared with the no use of AKI e-alerts, with substantial heterogeneity (RR, 0.96 [95% CI, 0.89-1.03]; *I*^2^ = 47% [95% CI, 0%-73%]) (Figure 1A). There was no significant subgroup difference of pooled effects between RCTs and non-RCTs.

In 5 studies,^10,11,12,20,25^ use of AKI e-alerts was associated with AKI stage progression (RR, 0.91 [95% CI, 0.84-0.99]), with substantial heterogeneity (*I*^2^ = 70% [95% CI, 23%-88%]). There was a significant subgroup difference of pooled effects between RCTs and non-RCTs (Figure 1B). All 5 studies reported AKI progression based on KDIGO criteria. Four studies defined AKI progression as advancing to a higher stage, except for Li et al.^12^ Three studies^10,11,12^ defined the period as in hospital, while 1 study^20^ defined it within 7 days.

Use of AKI e-alerts was associated with dialysis (RR, 1.16 [95% CI, 1.05-1.28]), with substantial heterogeneity (*I*^2^ = 50% [95% CI, 1%-75%]) and without significant subgroup differences (eFigure 4 in Supplement 1). Use of AKI e-alerts was associated with kidney recovery (RR, 1.13 [95% CI, 0.86-1.49]), with high heterogeneity (*I*^2^ = 98% [95% CI, 97%-99%]) and without significant subgroup difference (eFigure 4 in Supplement 1).

### Secondary Outcomes: Post-AKI Nephrologist Consultation, NSAID and ACEI/ARB Exposure, and Intravenous Fluid Prescription

**Figure 2.**
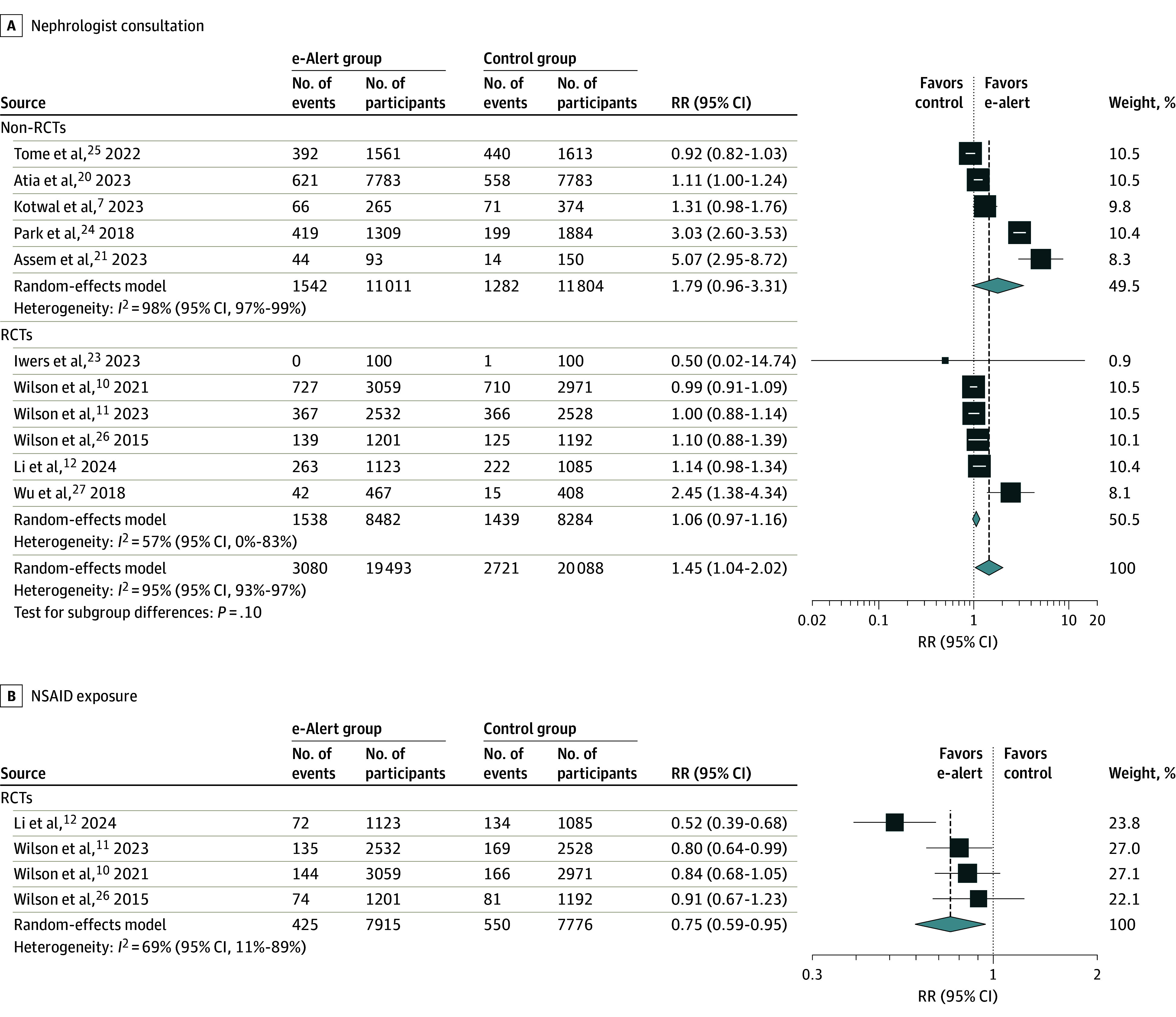
Association of Acute Kidney Injury (AKI) Electronic Alerts (e-Alerts) With Nephrologist Consultation and Nonsteroidal Anti-Inflammatory Drug (NSAID) Exposure RCT indicates randomized clinical trial; RR, risk ratio. Diamonds indicate heterogeneity; different marker sizes, weights.

Use of AKI e-alerts was associated with nephrologist consultation (RR, 1.45 [95% CI, 1.04-2.02]), with substantial heterogeneity (*I*^2^ = 95% [95% CI, 93%-97%]) and without significant subgroup difference (Figure 2A). Use of AKI e-alerts was associated with lower post-AKI NSAID exposure (RR, 0.75 [95% CI, 0.59-0.95]), with substantial heterogeneity (*I*^2^ = 69% [95% CI, 11%-89%]) (Figure 2B). The pooled RR for post-AKI ACEI/ARB exposure in the AKI e-alerts group compared with the control group was 0.91 (95% CI, 0.78-1.06), with substantial heterogeneity (*I*^2^ = 72% [95% CI, 19%-90%]) (eFigure 5 in Supplement 1). The pooled RR for post-AKI intravenous fluid prescription in the AKI e-alert group compared with the control group was 1.47 (95% CI, 0.86-2.54), with substantial heterogeneity (*I*^2^ = 97% [95% CI, 95%-98%]) and subgroup difference (eFigure 5 in Supplement 1).,

### Secondary Outcomes: Hospital LOS, Cost, and AKI Documentation

**Figure 3.**
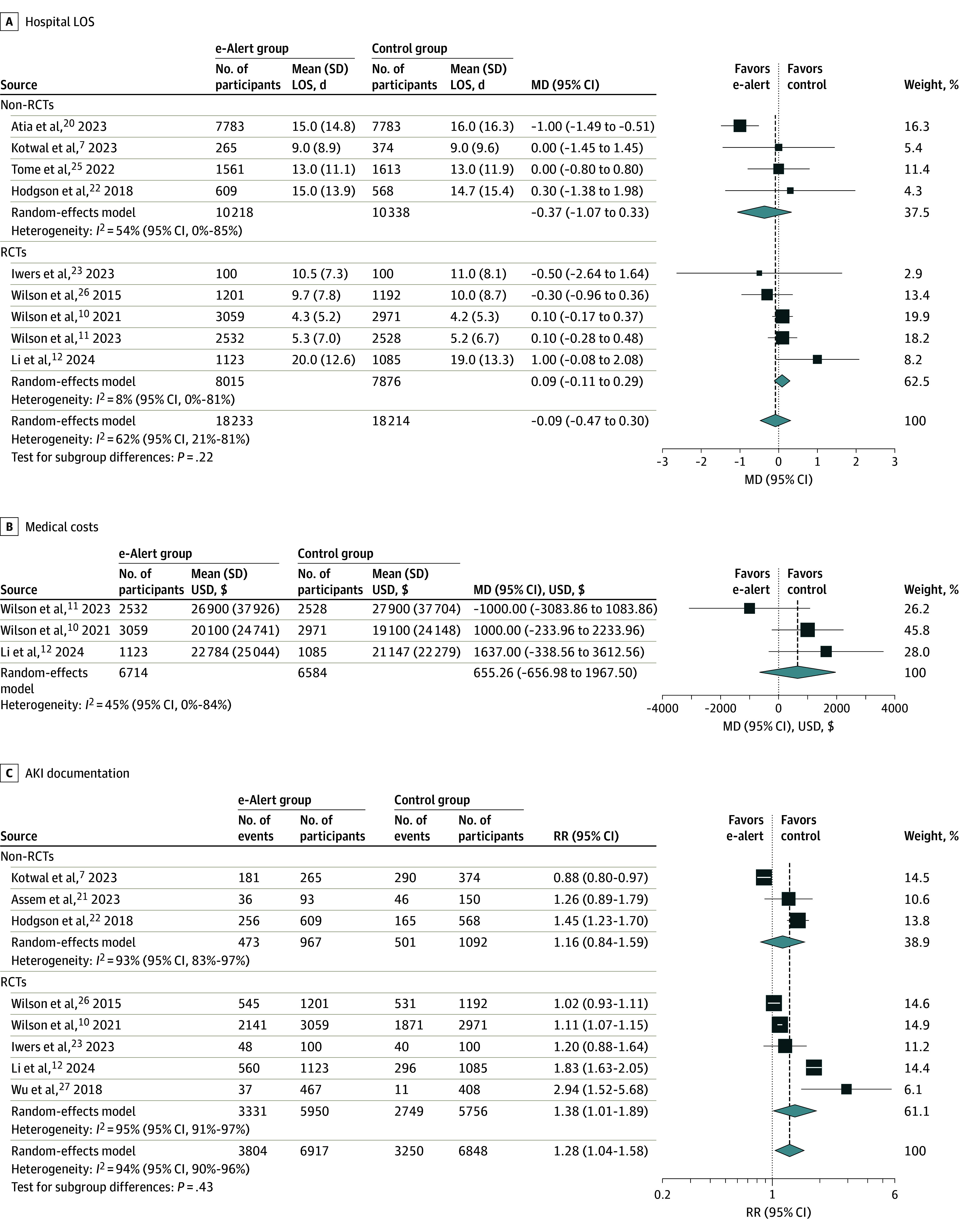
Association of Acute Kidney Injury (AKI) Electronic Alerts (e-Alerts) With Hospital Length of Stay (LOS), Medical Costs (B), and AKI Documentation (C) MD indicates mean difference; RCT, randomized clinical trial; RR, risk ratio; and USD, US dollars. Diamonds indicate heterogeneity; different marker sizes, weights.

Use of AKI e-alerts was not associated with lower hospital LOS compared with the control group, with a mean difference of −0.09 (95% CI, −0.47 to 0.30) days and substantial heterogeneity (*I*^2^ = 62% [95% CI, 21%-81%]), without significant subgroup difference (Figure 3A). Use of AKI e-alerts was not associated with lower cost compared with the control group, with a mean difference of US $655.26 (95% CI, −$656.98 to $1967.5) and low heterogeneity (*I*^2^ = 45% [95% CI, 0%-84%]) (Figure 3B). Use of AKI e-alerts was associated with greater AKI documentation (RR, 1.28 [95% CI, 1.04-1.58]), with substantial heterogeneity (*I*^2^ = 94% [95% CI, 90%-96%]) (Figure 3C).

### Subgroup Analysis

Subgroup analysis was performed by dividing enrolled studies into e-alerts in combination with an AKI care bundle or recommendation and those studies without. For AKI progression, studies with AKI e-alerts combined with AKI care bundle or recommendation had a lower RR compared with the non–e-alert groups (RR, 0.85 [95% CI, 0.77-0.93]; *P* = .03 for subgroup difference test) (eFigure 6 in Supplement 1). For the other 3 primary outcomes (mortality, dialysis, and kidney recovery) (eFigures 7-9 in Supplement 1) and most secondary outcomes (eFigures 10-15 in Supplement 1), there was no significant subgroup heterogeneity detected. Regarding post-AKI ACEI/ARB exposure, AKI e-alerts combined with an AKI care bundle were associated with lower RR (0.78 [95% CI, 0.70-0.88]; *P* = .002 for subgroup difference test) (eFigure 16 in Supplement 1). For studies reporting mortality outcomes over different time periods, there was no significant subgroup difference (eFigure 17 in Supplement 1).

### Trial Sequential Analysis and Sensitivity Analysis

A trial sequential analysis on mortality indicated that e-alerts were unlikely to be associated with a 10% risk reduction (eFigure 18 in Supplement 1). For AKI stage progression, TSA indicated a premature conclusion (eFigure 19 in Supplement 1). For dialysis, TSA show a true-positive finding with sufficient sample size (eFigure 20 in Supplement 1) and an uncertain result regarding kidney recovery (eFigure 21 in Supplement 1). Trial sequential analysis also supported the results from conventional analysis regarding nephrologist consultations, AKI documentation, and reduced post-AKI NSAID exposure (eFigures 22-24 in Supplement 1).

Including only RCTs in the TSA, e-alerts showed a true-positive finding for dialysis, NSAID exposure, and consultation (eTable 4 in Supplement 1). Other outcomes were premature, inconclusive, or ineffective. Sensitivity analysis using the Hartung-Knapp method still showed a significantly increased the RR for dialysis. The beta-binomial bayesian meta-analysis also showed a significantly lower RR for NSAID exposure after AKI (eTable 5 in Supplement 1).

### Publication Bias and Certainty of Evidence

The funnel plot for all primary and secondary outcomes are provided (eFigure 25 in Supplement 1). There was no significant asymmetry observed in the funnel plots. The Egger tests were performed for outcomes with more than 10 studies and found no publication bias for mortality (Egger *P* = .13), dialysis (Egger *P* = .63), or nephrologist consultation (Egger *P* = .26).

The overall certainty of evidence (CoE) varied from moderate to very low. We summarized the results of CoE assessment in eTable 6 in Supplement 1. The detailed reasons for downgrading are provided in eTable 6 in Supplement 1 and eAppendix 3 in Supplement 1. We also summarized the results and CoE assessment (Table 2).

**Table 2.** Summary of Finding Table

Outcomes	Anticipated absolute effects		RR (95% CI)	No. of participants	Certainty of the evidence (GRADE)
	Risk with control	Risk with AKI e-alert (95% CI)			
Mortality	167 per 1000	161 per 1000 (149 to 172)	0.96 (0.89- 1.03)	38 644	Low
AKI progression	190 per 1000	173 per 1000 (160 to 188)	0.91 (0.84- 0.99)	32 038	Moderate
Dialysis	59 per 1000	68 per 1000 (62 to 75)	1.16 (1.05- 1.28)	37 467	Very low
Kidney recovery	510 per 1000	577 per 1000 (439 to 760)	1.13 (0.86-1.49)	6519	Very low
Nephrologist consultation	135 per 1000	196 per 1000 (141 to 274)	1.45 (1.04-2.02)	39 581	Very low
NSAID exposure after AKI	71 per 1000	53 per 1000 (42 to 67)	0.75 (0.59-0.95)	15 691	Low
Hospital LOS	Mean, 11.9 d	MD −0.09 (−0.47 to 0.39) d	NA	36 447	Very low
Medical cost	Mean, US $22 816.19r	MD, $655.26 (−$656.98 to $1967.50)	NA	13 298	Moderate
AKI documentation	475 per 1000	607 per 1000 (494 to 750)	1.28 (1.04-1.58)	13 765	Very low
ACEI/ARB prescription	156 per 1000	142 per 1000 (121 to 165)	0.91 (0.78-1.06)	15 691	Moderate
Fluid prescription	437 per 1000	643 per 1000 (376 to 1000)	1.47 (0.86-2.54)	11 910	Very low

Abbreviations: ACEI/ARB, angiotensin-converting enzyme inhibitor and/or angiotensin receptor blocker; AKI, acute kidney injury; e-alert, electronic alert; GRADE, Grades of Recommendation, Assessment, Development, and Evaluation; LOS, length of stay; MD, mean difference; NA, nonapplicable; NSAID, nonsteroidal anti-inflammatory drug; RR, relative risks.

## Discussion

This systematic review and meta-analysis highlights 4 key findings. First, AKI e-alerts may be unlikely to be associated with a 10% reduction of risk for mortality in patients with AKI, a finding supported by TSA. Second, AKI e-alerts might be associated with lower RR of AKI progression, but more research is needed to support this conclusion. Third, AKI e-alerts were linked to increased dialysis events. Fourth, AKI e-alerts seem to be associated with different clinical practices (eg, more nephrologist consultations and AKI documentation and less post-AKI NSAID exposure).

Our analysis suggests AKI e-alerts may not be associated with lower RR of mortality but with lower RR of AKI progression, and the pooled effect was associated with heterogeneity. Most studies found AKI e-alerts have a neutral effect on mortality, with exceptions in 2 studies.^10,25^ Tome et al^25^ observed lower mortality with AKI e-alerts plus care recommendations in early-stage AKI, but not in stage 3. Conversely, the ELAIA-1 study^10^ noted increased mortality in nonteaching hospitals. The increased primary composite outcome from AKI e-alerts group in nonteaching hospitals was driven by increased mortality, but the dialysis or AKI progression rates were similar across different hospitals.^10^ In that study,^10^ the authors considered the harm from e-alerts in nonteaching hospitals to be a true effect and postulated several possible mechanisms, including unnecessary intravenous fluid prescription and/or fluid overload, alarm fatigue, and the pressure on clinicians to take potentially harmful actions (which could be prevented by systems in teaching hospitals).

The apparent neutrality, yet underlying heterogeneity, of AKI e-alerts’ association with mortality may result from several factors. First, evaluated AKI e-alert systems use creatinine-based diagnoses, with unreported variations in testing frequency and timing affecting AKI detection. Creatinine level, a delayed and less predictive AKI marker than urine output in patients with critical illness, could affect outcomes. Bianchi et al^28^ noted oliguria over 12 hours as a crucial outcome marker, independent of creatinine levels. Second, the AKI e-alert system’s success may depend more on alarm detection and management. The analysis by Shi et al^29^ showed physician response to and detection rate of e-alerts might result in different 14-day mortality rates. Third, reducing post-AKI NSAID exposure might explain the lower RR for AKI progression. As previously mentioned, the current limitations of AKI e-alerts may arise from the delayed nature of serum creatinine levels in AKI diagnosis and the heterogeneous effects of AKI e-alerts (which might be raised from different AKI care bundles, different hospitals, or physicians’ responses). Therefore, we suggest that an e-alert system should be integrated with earlier risk stratification methods, such as the renal angina index,^30,31^ artificial intelligence − based continuous AKI prediction,^32^ and care bundle implementation within a clinical decision support system to enhance early diagnosis and management, potentially improving outcomes.

Our analysis suggests that AKI e-alerts may increase post-AKI dialysis events, with more nephrologist consultations and reduced NSAID exposure. The higher dialysis rates in the e-alert group might result from the lack of standardized kidney replacement therapy initiation protocols and increased nephrologist involvement. Despite a lack of significant subgroup differences between RCTs and non-RCTs, 2 non-RCTs^20,21^ noted higher dialysis rates with e-alerts. Atia et al^20^ attributed this to more nephrologist consultations and earlier dialysis initiation. Our analysis found that the e-alert had lower RRs for post-AKI ACEI/ARB prescription. Only Wilson et al^11^reported the effect of e-alerts on post-AKI proton-pump inhibitor prescriptions. In that study, the e-alert group had a higher RR for proton-pump inhibitor therapy discontinuation (RR, 1.26 [95% CI, 1.10-1.45]).

Additionally, AKI e-alerts might reduce AKI stage progression, though with notable subgroup heterogeneity. Studies with AKI e-alerts plus care recommendations showed reduced AKI progression risk (eFigure 6 in Supplement 1). However, this finding, according to trial sequential analysis (eFigure 15 in Supplement 1), is premature, given the small number of studies reporting this outcome and the reliance on non-RCTs.

### Strengths and Limitations 

Our study has several strengths. It is an updated systematic review and meta-analysis on AKI e-alerts using contemporary evidence-based methods, includes trial sequential analysis, and evaluates the CoE across all outcomes using the GRADE framework. However, limitations exist. First, the scarcity of RCTs led us to combine findings from RCTs, prospective trials, and retrospective studies. Second, few studies examined the impact on hospital LOS, cost, AKI stage progression, post-AKI kidney recovery and ACEI/ARB prescription, limiting our ability to make conclusive statements on these aspects. Third, the evidence lacks exploration of urine output–based AKI e-alerts and early biomarkers for risk stratification, which could enhance early detection and intervention. Fourth, none of the enrolled studies reported major adverse kidney events at 28 and 90 days, which are crucial outcomes after AKI. This leaves the impact of AKI e-alerts and increased dialysis events on long-term outcomes uncertain. Further investigation in these areas is needed.

## Conclusions

The current meta-analysis suggests that the implementation of AKI e-alerts might not be associated with a lower risk for mortality but may be associated with different practice patterns (including higher RRs for nephrologist consultations, dialysis, and AKI documentation and lower RRs for post-AKI NSAID exposure). Implementation was associated with a lower RR for AKI progression, but this result was heterogeneous and possibly premature. We recommend that each hospital establish its own AKI e-alert system and individualized AKI management protocol tailored to its specific needs. Additionally, future studies should focus on combining e-alert systems with AKI prediction or early biomarker risk stratification, along with clinical decision support systems or care bundles, which might be beneficial.

## Notes

Supplement 1. **eTable 1.** Search Strategy and Result **eTable 2.** Reasons for Excluding Full-Text Screening Studies **eTable 3.** Inclusion and Exclusion Criteria and Care Bundle or Suggestions for Enrolled Studies **eTable 4.** Trial Sequential Analysis With Only Enrolled Randomized Clinical Trials **eTable 5.** Sensitivity Analysis **eTable 6.** Summary of Certainty of Evidence Assessment **eFigure 1.** PRISMA Flow Diagram **eFigure 2.** Version 2 of the Cochrane Risk-of-Bias Tool for Randomized Trials (RoB 2.0) Assessment of Included Studies and Summary **eFigure 3.** Risk of Bias in Nonrandomized Studies of Interventions (ROBINS-I) Assessment of Included Studies and Summary **eFigure 4.** Forest Plot Illustrating the Association of Acute Kidney Injury (AKI) Electronic Alerts (e-Alerts) With Dialysis and Kidney Recovery after AKI **eFigure 5.** Forest Plot Illustrating the Association of Acute Kidney Injury (AKI) Electronic Alerts (e-Alerts) With ACEI/ARB Prescription and Fluid Prescription After AKI **eFigure 6.** Subgroup Analysis for AKI Progression **eFigure 7.** Subgroup Analysis for Mortality **eFigure 8.** Subgroup Analysis for Dialysis **eFigure 9.** Subgroup Analysis for Kidney Recovery **eFigure 10.** Subgroup Analysis for Nephrologist Consultation **eFigure 11.** Subgroup Analysis for NSAID Exposure After AKI **eFigure 12.** Subgroup Analysis for Hospital Length of Stay **eFigure 13.** Subgroup Analysis for Medical Costs **eFigure 14.** Subgroup Analysis for AKI Documentation **eFigure 15.** Subgroup Analysis for Fluid prescription **eFigure 16.** Subgroup Analysis for ACEI/ARB exposure **eFigure 17.** Subgroup Analysis for Mortality With Different Follow-Up Period **eFigure 18.** Trial Sequential Analysis for Mortality **eFigure 19.** Trial Sequential Analysis for AKI Progression **eFigure 20.** Trial Sequential Analysis for Dialysis **eFigure 21.** Trial Sequential Analysis for Kidney Recovery **eFigure 22.** Trial Sequential Analysis for Nephrologist Consultation **eFigure 23.** Trial Sequential Analysis for NSAID Exposure After AKI **eFigure 24.** Trial Sequential Analysis for AKI documentation **eFigure 25.** Funnel Plots **eAppendix 1.** Supplemental Method for Trial Sequential Analysis **eAppendix 2.** Quality of Included Studies **eAppendix 3.** Certainty of Evidence Assessment for AKI Progression

Supplement 2.
Data Sharing Statement
