# Supplementary material for: Electronic Alert Systems for Patients With Acute Kidney Injury: A Systematic Review and Meta-Analysis
Source: JAMA Netw Open. 2024 Aug 27;7(8):e2430401. doi: 10.1001/jamanetworkopen.2024.30401 (PMC11350470; doi:10.1001/jamanetworkopen.2024.30401)
Supplement: Supplement 1. — eTable 1. Search Strategy and Result eTable 2. Reasons for Excluding Full-Text Screening Studies eTable 3. Inclusion and Exclusion Criteria and Care Bundle or Suggestions for Enrolled Studies eTable 4. Trial Sequential Analysis With Only Enrolled Randomized Clinical Trials eTable 5. Sensitivity Analysis eTable 6. Summary of Certainty of Evidence Assessment eFigure 1. PRISMA Flow Diagram eFigure 2. Version 2 of the Cochrane Risk-of-Bias Tool for Randomized Trials (RoB 2.0) Assessment of Included Studies and Summary eFigure 3. Risk of Bias in Nonrandomized Studies of Interventions (ROBINS-I) Assessment of Included Studies and Summary eFigure 4. Forest Plot Illustrating the Association of Acute Kidney Injury (AKI) Electronic Alerts (e-Alerts) With Dialysis and Kidney Recovery after AKI eFigure 5. Forest Plot Illustrating the Association of Acute Kidney Injury (AKI) Electronic Alerts (e-Alerts) With ACEI/ARB Prescription and Fluid Prescription After AKI eFigure 6. Subgroup Analysis for AKI Progression eFigure 7. Subgroup Analysis for Mortality eFigure 8. Subgroup Analysis for Dialysis eFigure 9. Subgroup Analysis for Kidney Recovery eFigure 10. Subgroup Analysis for Nephrologist Consultation eFigure 11. Subgroup Analysis for NSAID Exposure After AKI eFigure 12. Subgroup Analysis for Hospital Length of Stay eFigure 13. Subgroup Analysis for Medical Costs eFigure 14. Subgroup Analysis for AKI Documentation eFigure 15. Subgroup Analysis for Fluid prescription eFigure 16. Subgroup Analysis for ACEI/ARB exposure eFigure 17. Subgroup Analysis for Mortality With Different Follow-Up Period eFigure 18. Trial Sequential Analysis for Mortality eFigure 19. Trial Sequential Analysis for AKI Progression eFigure 20. Trial Sequential Analysis for Dialysis eFigure 21. Trial Sequential Analysis for Kidney Recovery eFigure 22. Trial Sequential Analysis for Nephrologist Consultation eFigure 23. Trial Sequential Analysis for NSAID Exposure After AKI eFigure 24. Trial Sequential Analysis for [file jamanetwopen-e2430401-s001.pdf]

Supplementary Online Content

Chen J-J, Lee T-H, Chan M-J, et al. Electronic alert systems for patients with acute kidney injury: a systematic review and meta-analysis. *JAMA Netw Open*. 2024;7(8):e2430401.
doi:10.1001/jamanetworkopen.2024.30401

eTable 1. Search Strategy and Result

eTable 2. Reasons for Excluding Full-Text Screening Studies

eTable 3. Inclusion and Exclusion Criteria and Care Bundle or Suggestions for Enrolled Studies

eTable 4. Trial Sequential Analysis With Only Enrolled Randomized Clinical Trials

eTable 5. Sensitivity Analysis

eTable 6. Summary of Certainty of Evidence Assessment

eFigure 1. PRISMA Flow Diagram

eFigure 2. Version 2 of the Cochrane Risk-of-Bias Tool for Randomized Trials (RoB 2.0) Assessment of Included Studies and Summary

eFigure 3. Risk of Bias in Nonrandomized Studies of Interventions (ROBINS-I) Assessment of Included Studies and Summary

eFigure 4. Forest Plot Illustrating the Association of Acute Kidney Injury (AKI) Electronic Alerts (e-Alerts) With Dialysis and Kidney Recovery after AKI

eFigure 5. Forest Plot Illustrating the Association of Acute Kidney Injury (AKI) Electronic Alerts (e-Alerts) With ACEI/ARB Prescription and Fluid Prescription After AKI

eFigure 6. Subgroup Analysis for AKI Progression

eFigure 7. Subgroup Analysis for Mortality

eFigure 8. Subgroup Analysis for Dialysis

eFigure 9. Subgroup Analysis for Kidney Recovery

© 2024 Chen JJ et al. *JAMA Network Open*.

eFigure 10. Subgroup Analysis for Nephrologist Consultation

eFigure 11. Subgroup Analysis for NSAID Exposure After AKI

eFigure 12. Subgroup Analysis for Hospital Length of Stay

eFigure 13. Subgroup Analysis for Medical Costs

eFigure 14. Subgroup Analysis for AKI Documentation

eFigure 15. Subgroup Analysis for Fluid prescription

eFigure 16. Subgroup Analysis for ACEI/ARB exposure

eFigure 17. Subgroup Analysis for Mortality With Different Follow-Up Period

eFigure 18. Trial Sequential Analysis for Mortality

eFigure 19. Trial Sequential Analysis for AKI Progression

eFigure 20. Trial Sequential Analysis for Dialysis

eFigure 21. Trial Sequential Analysis for Kidney Recovery

eFigure 22. Trial Sequential Analysis for Nephrologist Consultation

eFigure 23. Trial Sequential Analysis for NSAID Exposure After AKI

eFigure 24. Trial Sequential Analysis for AKI documentation

eFigure 25. Funnel Plots

eAppendix 1. Supplemental Method for Trial Sequential Analysis

eAppendix 2. Quality of Included Studies

eAppendix 3. Certainty of Evidence Assessment for AKI Progression

This supplementary material has been provided by the authors to give readers additional information about their work.

© 2024 Chen JJ et al. *JAMA Network Open*.

eTable 1. Search Strategy and Result

**Pubmed through March 18st, 2024**	
#1	Electronic alert
#2	eAlert
#3	acute kidney injury
#4	acute renal failure
#5	(acute renal failure) OR (acute kidney injury)
#6	(eAlert) OR (Electronic alert)
#7	((eAlert) OR (Electronic alert)) AND ((acute renal failure) OR (acute kidney injury)), Result: 189
**EMbase through March 18st, 2024**	
#1	('acute kidney failure'/exp OR 'acute kidney injury') AND ('electronic alert'/exp OR ealert), Result: 98
**Cochrane library through March 20st, 2024**	
#1	(acute kidney injury):ti,ab,kw
#2	("acute renal failure"):ti,ab,kw
#3	(acute kidney failure):ti,ab,kw
#4	MeSH descriptor: [Acute Kidney Injury] explode all trees
#5	#1 OR #2 OR #3 OR #4
#6	(electronic alert):ti,ab,kw
#7	(eAlert):ti,ab,kw
#8	(e-Alert):ti,ab,kw
#9	#6 OR #7 OR #8
#10	#5 AND #9 , Result 42 articles

© 2024 Chen JJ et al. *JAMA Network Open*.

eTable 2. Reasons for Excluding Full-Text Screening Studies

Number	First author, Year	Title	Reason for exclusion
1	Halmy, 2021	Renal Recovery after the Implementation of an Electronic Alert and Biomarker-Guided Kidney-Protection Strategy following Major Surgery	e-Alert according to biomarker not clinical AKI (Only 32% AKI)
2	Biswas, 2018	Identification of Patients Expected to Benefit from Electronic Alerts for Acute Kidney Injury	Duplication
3	Shi, 2022, 36466503	The rate of acute kidney injury (AKI) alert detection by the attending physicians was associated with the prognosis of patients with AKI	
4	Barker, 2021	Electronic alerts for acute kidney injury across primary and secondary care	Lack of AKI patient number
5	Chen-Xu, 2024	Impact of electronic AKI alert/care bundle on AKI inpatient outcomes: a retrospective single-center cohort study	
6	Selby, 2019	An Organizational-Level Program of Intervention for AKI: A Pragmatic Stepped Wedge Cluster Randomized Trial	
7	Barton, 2020	Acute Kidney Injury in Primary Care: A Review of Patient Follow-Up, Mortality, and Hospital Admissions following the Introduction of an AKI Alert System	No controlled group
8	Haase-Fielitz, 2020	The Effects of Intensive Versus Routine Treatment in Patients with Acute Kidney Injury	
9	Holmes, 2016	Acute Kidney Injury in the Era of the AKI E-Alert	
10	Holmes, 2018	Utility of electronic AKI alerts in intensive care: A national multicentre cohort study	
11	Holmes, 2021	Acute kidney injury demographics and outcomes: changes following introduction of electronic acute kidney injury alerts—an analysis of a national dataset	
12	Kolhe, 2016	A simple care bundle for use in acute kidney injury: a propensity score-matched cohort study	

© 2024 Chen JJ et al. *JAMA Network Open*.

13	Thomas, 2021	The Acute Kidney Outreach to Prevent Deterioration and Death trial: a large pilot study for a cluster-randomized trial	
14	Aiyegbusi, 2018	Impact of introducing electronic acute kidney injury alerts in primary care	No outcome of interest
15	Niemantsverdriet, 2023	Design, validation and implementation of an automated e-alert for acute kidney injury: 6-month pilot study shows increased awareness	
16	Purvis, 2018,	DEVELOPMENT AND IMPLEMENTATION OF AN ACUTE KIDNEY INJURY E-ALERT SYSTEM IN ACUTE MEDICAL RECEIVING UNITS IN GLASGOW	
17	Pyart, 2016	Acute Kidney Injury Electronic Alert Triggered Intervention Improves Length of Stay	
18	Qian, 2021	ACUTE KIDNEY INJURY (AKI) ELECTRONIC ALERTS AND AKI CARE BUNDLE PROJECT	
19	Tollitt, 2018,	Improved management of acute kidney injury in primary care using e-alerts and an educational outreach programme	
20	West Midlands Acute Medicine Collaborative, 2019	The impact of the NHS electronic-alert system on the recognition and management of acute kidney injury in acute medicine	
21	Moran, 2015	Acute Kidney Injury: Adding Informatics to Injury – (Electronic Injury Alerts)	No AKI event number

© 2024 Chen JJ et al. *JAMA Network Open*.

eTable 3. Inclusion and Exclusion Criteria and Care Bundle or Suggestions for Enrolled Studies

Study	Inclusion criteria	Exclusion criteria	AKI care bundle or suggestion
Atia et al,[21] 2023	NR	Patients who were already on a dialysis	(1) Senior clinical review<br>(2) Follow up Trust AKI guideline and care bundle
Assem et al,[22] 2023	NR	NR	(1) Evaluate volume status<br>(2) Discontinue NSAIDs/ACEI/ARB<br>(3) Consider alternatives to radiocontrast procedures<br>(4) Review aminoglycoside indication<br>(5) Check for changes in drugs dosing<br>(6) Review antihypertensive dose<br>(7) Avoid hyperglycemia<br>(8) Request creatinine daily<br>(9) Complete fluid chart<br>(10) Monitor volume output<br>(11) Request urinalysis / Renal ultrasound
Colpaert et al,[8] 2012	All patients admitted to the 36-bed surgical and medical ICU were screened for inclusion	<18 years old, chronic kidney disease stage 5 or on dialysis, renal transplantation within the last 3 months, nephrectomy, or recent kidney trauma	Not included

© 2024 Chen JJ et al. *JAMA Network Open*.

Hodgson et al,[23] 2018	>18 years of age, stayed at least one night in the medical unit and had at least one SCr repeated	Patients moved directly to the ICU or aged <18 years or Non-medical (General Surgery, Trauma & Orthopaedics, Obstetrics and Gynaecology) admissions	(1) Search for underlying cause of admission<br>(2) Fluid bolus & re-assess, fluid balance chart, daily weights<br>(3) Review drug chart & stop potential nephrotoxic<br>(4) Urine dip 6 hour<br>(5) Re-review fluid status, consider escalation of care<br>(6) Where appropriate consider imaging renal tract<br>(7) Consider other specific blood tests: autoimmune, CK<br>(8) If not improving for discussion with Nephrology
Iwers et al,[24] 2023	Admitted to the Department of Cardiology with AKI (KDIGO criteria), be over 18 years of age, and be able to sign a written informed consent form	Patients who had already undergone kidney transplantation or who were on dialysis, pregnant patients, patients with HIV/Hepatitis virus infections, or patients with a life expectancy below 3 months	(1) Identification of the cause(s) of AKI in the clinical context<br>(2) Measures to achieve euvolemia (through fluid administration or negative balance)<br>(3) Pharmacological intervention including discontinuation of nephrotoxic drugs or switch to less nephrotoxic drugs of the same substance class or monitoring of plasma levels<br>(4) Adjustment of drug doses to renal function<br>(5) Optimizing hemodynamics<br>(6) Detection, and treatment of electrolyte and acid-base disorders

© 2024 Chen JJ et al. *JAMA Network Open*.

			(7) Monitoring of heart and kidney function during the index hospital stay<br>(8) Nephrology consultation of patients with AKI stage 3
Kotwal et al,[7] 2023	All patients admitted to the three hospitals, developed AKI identified by an automated algorithm using serum creatinine measurements, were aged ≥18 years	Patients with end-stage kidney disease on dialysis, new kidney transplant recipients and pregnant patients	STOP AKI assessment plan (Sepsis, Toxin, Optimise blood pressure, Prevent harm)
Li et al,[12] 2024	The inclusion criteria were inpatient adults 18 years or older with AKI	Baseline estimated glomerular filtration rate lower than 15 mL/min/1.73m2, admission diagnosis of end-stage kidney disease, history of kidney transplant, AKI occurring outside the hospital, hospitalization for less than 24 hours, and baseline SCr levels lower than 0.5 mg/dL	General, non-individualized, and nonmandatory AKI management measures:<br>(1) AKI requires optimization of hemodynamics<br>(2) Discontinuation of unnecessary nephrotoxic drugs<br>(3) Adjustment of antimicrobial drug dosage<br>(4) Dialysis if necessary<br>(5) For diagnosis and treatment inquiries, please contact the nephrology consultation service
Park et al,[25] 2018	Patients who had AKI in the study cohort were included in the alert group, and those with AKI events in the historical cohort, identified based on the same criteria used for the alert group, were included in the usual-care group	(1) ongoing renal replacement therapy, (2) impending end-stage renal disease (baseline estimated glomerular filtration rate < 15 mL/min/ 1.73 m2), (3) admission to the nephrology division because these patients were already receiving care from the attending	Not included

© 2024 Chen JJ et al. *JAMA Network Open*.

		nephrologists, (4) death events on the day of AKI development, and (5) patients who were already enrolled in the historical cohort	
Tome et al,[26] 2022	Patients aged ≥18 years	Patients admitted to the nephrology department and palliative care wards with end-stage renal disease or basal serum creatinine levels of >4.0 mg/dL	(1) Diagnosis: STOP (sepsis, toxicology, obstruction, parenchymal kidney disease)<br>(2) Recommendation:<br>A. Evaluate volume status<br>B. Discontinue NSAID/ACEI/ARB<br>C. Consider alternatives to radiocontrast procedure<br>D. Review aminoglycoside indication<br>E. Check for changes in drug dosing<br>F. Review antihypertensive dose<br>G. Avoid hyperglycemia<br>H. Request creatinine daily /complete fluid chart<br>I. Monitor volume output<br>J. Request urine analysis/ renal ultrasound<br>(3) Nephrologist consultation
Wilson et al,[27] 2015	Adults aged 18 years or older who were in hospital with acute kidney injury as defined by the KDIGO creatinine- based criteria	Initial hospital creatinine 4.0 mg/dL or greater, fewer than two creatinine values measured, inability to determine the covering provider, admission to hospice or the observation unit,	Not included

© 2024 Chen JJ et al. *JAMA Network Open*.

		previous randomization, or admission International Classification of Diseases-9 code 58·6 (end-stage renal disease).	
Wilson et al,[10] 2021	Inpatient adults aged 18 or older with acute kidney injury, defined by the Kidney Disease: Improving Global Outcomes (KDIGO) criteria as an increase in creatinine 0.3 mg/dL (26.5 μmol/L) within 48 hours or 1.5 times the lowest measured creatinine within the previous seven days of admission to hospital,	Patients with a history of end stage kidney disease (based on International Classification of Disease, ninth and 10th revision (ICD-9 and ICD- 10) codes, a dialysis order in the past year, or an initial creatinine >4 mg/dL, whose admission date was before the inception of alerts at a study hospital, whose first alert occurred after hospital discharge, who had been enrolled in a previous study, and who were enrolled during a two week period in which alerting ceased owing to an upgrade of the electronic health record system	Not included
Wilson et al,[11] 2023	Adults >= 18 years old, who had AKI as defined by the KDIGO serum creatinine criteria, and had an active order for one or more of the three MOIs (NSAIDs; RAASi and PPIs)	Initial creatinine >= 4.0 mg/dL or if they had received dialysis within the year prior to meeting the AKI definition, if they had been admitted to a hospice service or had an active “comfort measures only” order, if they had a diagnosis code consistent with end-stage kidney disease, or if they had a kidney	Consider clinical indication for the following medications

© 2024 Chen JJ et al. *JAMA Network Open*.

		transplant within the 6 months prior to randomization (as transplant recipients at our institution receive specialist nephrology care during their inpatient stay, regardless of AKI)	
Wu et al,[28] 2018	Adult patients with an Alert for AKI(based on KDIGO guidelines)	Patients already on dialysis for AKI at the time of alert or patients with End stage renal disease or patients <18 years of age	Not included

Abbreviation: AKI, acute kidney injury; Cr, creatinine, KDIGO, Kidney Disease: Improving Global Outcomes; NR, not reported

© 2024 Chen JJ et al. *JAMA Network Open*.

eTable 4. Trial Sequential Analysis With Only Enrolled Randomized Clinical Trials

Outcome	Conventional boundary	Monitoring efficacy boundary	Futility boundary	Required information size	Interpretation
Mortality	Crossed	Not crossed	Crossed	17051, Nearly reached	10% RRR unlikely
AKI progression	Not crossed	Not crossed	Crossed	21143, Not reached	9% RRR unlikely
Dialysis	Crossed	Crossed	Not crossed	17158, Near reached	16% RRR likely (increased dialysis)
Recovery	Not crossed	Not crossed	Not crossed	6610, Not reached	Inconclusive
NSAID exposure[a]	Crossed	Crossed	Not crossed	19713, Nearly reached	25% RRR likely
Nephrologist consultation	Crossed	Crossed	Not crossed	2636, reached	45% RRR likely (increased dialysis)
AKI documentation	Crossed	Not crossed	Not crossed	24236, Not reached	Premature

Abbreviation: RRR, relative risk reduction

Footnote: [a]All RCT, similar to primary TSA

© 2024 Chen JJ et al. *JAMA Network Open*.

eTable 5. Sensitivity Analysis

**Outcome**	**Conventional DerSimonian and Laird method meta-analysis (RR, 95% CI)**	**Hartung-Knapp-Sidik-Jonkman approach (RR, 95% CI)**	**Beta-binomial Bayesian meta-analysis (Median, 95% Credible Interval)**
Mortality	0.96 (0.89 to 1.03)	0.96 (0.88 to 1.04)	0.945 (0.823 to 1.1)
AKI progression	0.91 (0.84 to 0.99)	0.91 (0.81 to 1.02)	0.903 (0.788 to 1.048)
Dialysis	1.16 (1.05 to 1.28)	1.157 (1.001 to 1.336)	1.153 (0.991 to 1.339)
Recovery	1.13 (0.86 to 1.49)	1.13 (0.74 to 1.74)	1.122 (0.465 to 1.744)
NSAID exposure after AKI	0.75 (0.59 to 0.95)	0.75 (0.51 to 1.11)	0.757 (0.6 to 0.94)
Nephrologist consultation	1.45 (1.04 to 2.02)	1.45 (0.99 to 2.12)	1.388 (0.386 to 3.94)
ACEI/ARB exposure after AKI	0.91 (0.78 to1.06)	0.91 (0.73 to 1.14)	0.901 (0.726 to 1.115)
AKI documentation	1.28 (1.04 to 1.58)	1.28 (0.98 to 1.68)	1.199 (0.707 to 2.144)
Intravenous fluid prescription after AKI	1.47 (0.86 to 2.54)	1.47 (0.68 to 3.18)	1.408 (0.389 to 3.398)

Abbreviation: AKI, acute kidney injury; ACEI/ARB, angiotensin converting enzyme inhibitor/angiotensin-receptor blocker; CI, confidence interval; NSAID, non-steroidal anti-inflammatory drug; RR, Risk Ratio

© 2024 Chen JJ et al. *JAMA Network Open*.

eTable 6. Summary of Certainty of Evidence Assessment

Certainty assessment							№ of patients		Effect		Certainty	Importance
**№ of studies**	**Study design**	**Risk of bias**	**Inconsistency**	**Indirectness**	**Imprecision**	**Other considerations**	**AKI eAlert**	**control**	**Relative (95% CI)**	**Absolute (95% CI)**		
**Mortality**												
12	non-randomised studies & randomised trials	very serious [a]	not serious	not serious	not serious	none	3041/19409 (15.7%)	3220/19235 (16.7%)	**RR 0.96** (0.89 to 1.03)	**7 fewer per 1,000** (from 18 fewer to 5 more)	⨁⨁◯◯ Low	IMPORTANT
**AKI progression**												
5	non-randomised studies & randomised trials	not serious [b]	not serious	not serious	serious[c]	none	2707/16058 (16.9%)	3038/15980 (19.0%)	**RR 0.91** (0.84 to 0.99)	**17 fewer per 1,000** (from 30 fewer to 2 fewer)	⨁⨁⨁◯ Moderate	IMPORTANT
**Dialysis**												

© 2024 Chen JJ et al. *JAMA Network Open*.

Certainty assessment							№ of patients		Effect		Certainty	Importance
№ of studies	Study design	Risk of bias	Inconsistency	Indirectness	Imprecision	Other considerations	AKI eAlert	control	Relative (95% CI)	Absolute (95% CI)		
11	non-randomised studies & randomised trials	very serious[d]	serious[e]	not serious	not serious[f]	none	1266/18800 (6.7%)	1098/18667 (5.9%)	**RR 1.16** (1.05 to 1.28)	**9 more per 1,000** (from 3 more to 16 more)	⨁◯◯◯ Very low	IMPORTANT
**Kidney recovery**												
4	non-randomised studies & randomised trials	very serious[g]	serious[h]	not serious	serious[i]	none	1949/2992 (65.1%)	1800/3527 (51.0%)	**RR 1.13** (0.86 to 1.49)	**66 more per 1,000** (from 71 fewer to 250 more)	⨁◯◯◯ Very low	IMPORTANT
**nephrologist consultation**												
11	non-randomised studies & randomised trials	very serious[j]	serious[k]	not serious	not serious	none	3080/19493 (15.8%)	2721/20088 (13.5%)	**RR 1.45** (1.04 to 2.02)	**61 more per 1,000** (from 5 more to 138 more)	⨁◯◯◯ Very low	IMPORTANT
**NSAID exposure after AKI**												

© 2024 Chen JJ et al. *JAMA Network Open*.

Certainty assessment							№ of patients		Effect		Certainty	Importance
№ of studies	Study design	Risk of bias	Inconsistency	Indirectness	Imprecision	Other considerations	AKI eAlert	control	Relative (95% CI)	Absolute (95% CI)		
4	randomised trials	not serious	serious[l]	not serious	not serious	none	425/7915 (5.4%)	550/7776 (7.1%)	**RR 0.75** (0.59 to 0.95)	**18 fewer per 1,000** (from 29 fewer to 4 fewer)	⊕⊕⊕◯ Moderate	IMPORTANT
**Hospital length of stay**												
9	non-randomised studies & randomised trials	very serious[m]	serious[n]	not serious	not serious	none	18233	18214	-	MD **0.09 days lower** (0.47 lower to 0.39 higher)	⊕◯◯◯ Very low	NOT IMPORTANT
**Medical cost**												
3	randomised trials	not serious	not serious	not serious	not serious	none	6714	6584	-	MD **655.26 dollor higher** (656.98 lower to 1967.5 higher)	⊕⊕⊕⊕ High	NOT IMPORTANT
**AKI documentation**												

© 2024 Chen JJ et al. *JAMA Network Open*.

Certainty assessment							№ of patients		Effect		Certainty	Importance
№ of studies	Study design	Risk of bias	Inconsistency	Indirectness	Imprecision	Other considerations	AKI eAlert	control	Relative (95% CI)	Absolute (95% CI)		
8	non-randomised studies & randomised trials	serious[o]	serious[p]	not serious	not serious	none	3804/6917 (55.0%)	3250/6848 (47.5%)	RR 1.28 (1.04 to 1.58)	133 more per 1,000 (from 19 more to 275 more)	⨁◯◯◯ Very low	IMPORTANT
**ACEI/ARB prescription**												
4	randomised trials	not serious	not serious[q]	not serious	serious[r]	none	1104/7915 (13.9%)	1211/7776 (15.6%)	RR 0.91 (0.78 to 1.06)	14 fewer per 1,000 (from 34 fewer to 9 more)	⨁⨁⨁◯ Moderate	IMPORTANT
**Fluid prescription**												
5	non-randomised studies	serious[s]	serious[t]	not serious	not serious	none	3271/6099 (53.6%)	2541/5811 (43.7%)	RR 1.47 (0.86 to 2.54)	206 more per 1,000 (from 61 fewer to 673 more)	⨁◯◯◯ Very low	IMPORTANT

© 2024 Chen JJ et al. *JAMA Network Open*.

Abbreviation: AKI, acute kidney injury; ACEI/ARB, angiotensin converting enzyme inhibitor/angiotensin-receptor blocker; CI, confidence interval; MD, mean difference; NSAID, non-steroidal anti-inflammatory drug; RR, Risk Ratio

Footnote/Explanations

a. 2 enrolled non-RCTs are high concern with risk of bias (overall 2 of 12) and 4 of 12 (33.3%; > 25%) have moderate risk of bias
b. 1 of the enrolled study is moderate risk and other 4 are low risk (>75% low risk)
c. The TSA demonstrated a premature conclusion
d. 2 enrolled studies (Assem, 2023; Iwers, 2023) have high risk of bias and only 3 of 11 enrolled studies (27.3%; > 25%) are low risk
e. I^2 50% and two pre-defined subgroup analysis did not demonstrated significant subgroup treatment effect heterogeneity
f. The TSA demonstrated and a RRR of 16% might be true result with sufficient sample size
g. 2 of 4 enrolled studies have high risk of bias (Assem, 2023; Park, 2018)
h. I^2 98% and two pre-defined subgroup analysis did not demonstrated significant subgroup treatment effect heterogeneity
i. Inconclusive result from both conventional meta-analysis and TSA
j. 3 of 11 enrolled studies have high risk of bias and 5 of 11 studies (45.5%; < 75%) have low risk of bias
k. I^2 96% and two pre-defined subgroup analysis did not demonstrated significant subgroup treatment effect heterogeneity
l. I^2 71% and two pre-defined subgroup analysis did not demonstrated significant subgroup treatment effect heterogeneity
m. 2 of 9 enrolled studies have high risk of bias and 5 of 9 studies (55.6%; < 75%) have low risk of bias
n. I^2 62% and two pre-defined subgroup analysis did not demonstrated significant subgroup treatment effect heterogeneity
o. 4 of 8 enrolled studies have high risk of bias
p. I^2 94% and two pre-defined subgroup analysis did not demonstrated significant subgroup treatment effect heterogeneity
q. I^2 72% and pre-defined subgroup analysis did demonstrated significant subgroup treatment effect heterogeneity (trials with care bundle vs. trials without non care bundle)
r. A pre-specified RR of 0.9 to 1.1 was considered to indicate no effect. The 95% CI crossed both areas of significant effect and no effect.
s. 2 of 5 enrolled studies have not low risk of bias
t. I^2 97% but pre-defined subgroup analysis did demonstrated significant subgroup treatment effect heterogeneity

© 2024 Chen JJ et al. *JAMA Network Open*.

eFigure 1. PRISMA Flow Diagram

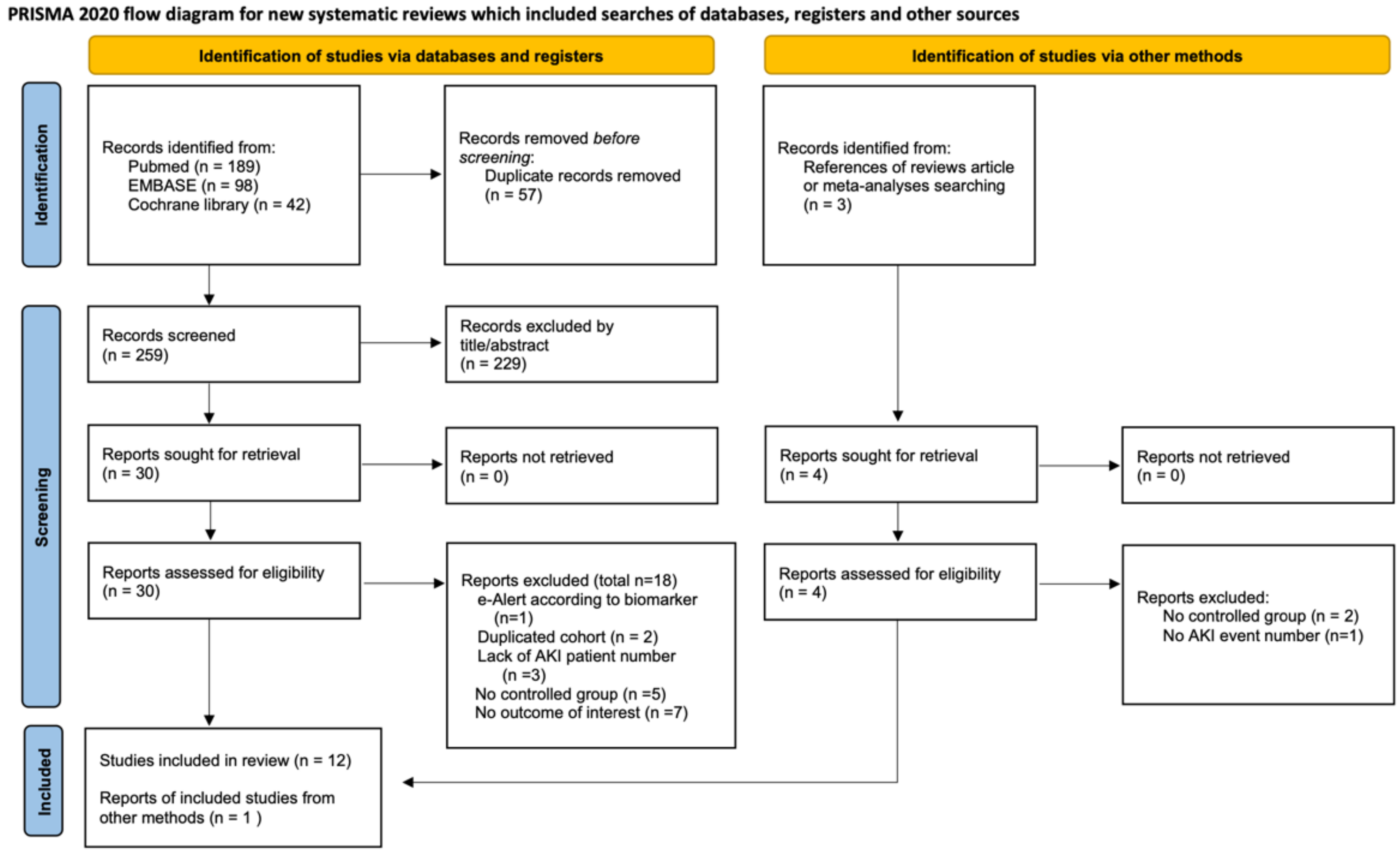

© 2024 Chen JJ et al. *JAMA Network Open*.

eFigure 2. Version 2 of the Cochrane Risk-of-Bias Tool for Randomized Trials (RoB 2.0) Assessment of Included Studies and Summary

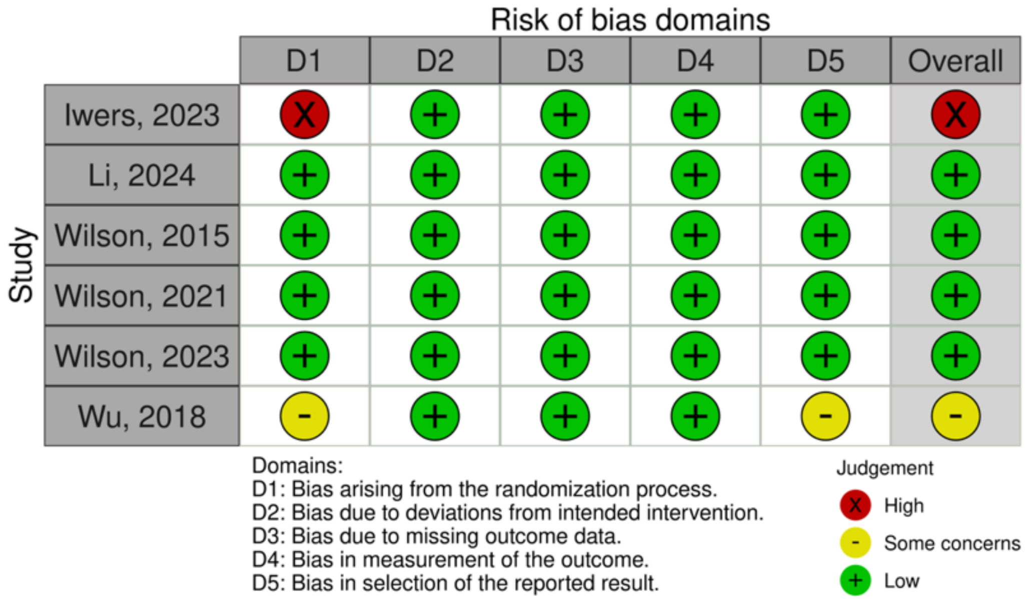

© 2024 Chen JJ et al. *JAMA Network Open*.

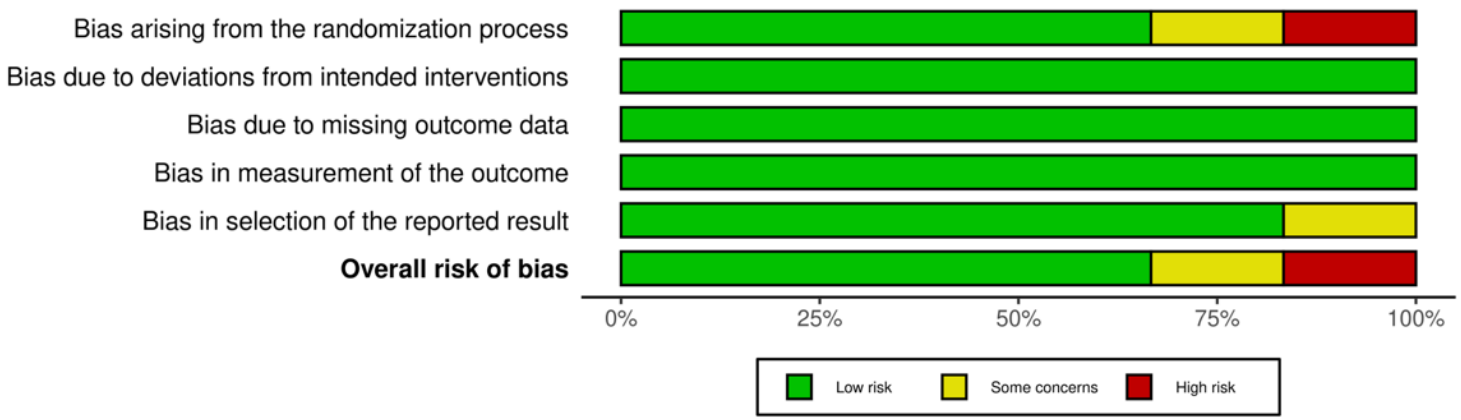

© 2024 Chen JJ et al. *JAMA Network Open*.

eFigure 3. Risk of Bias in Nonrandomized Studies of Interventions (ROBINS-I) Assessment of Included Studies and Summary

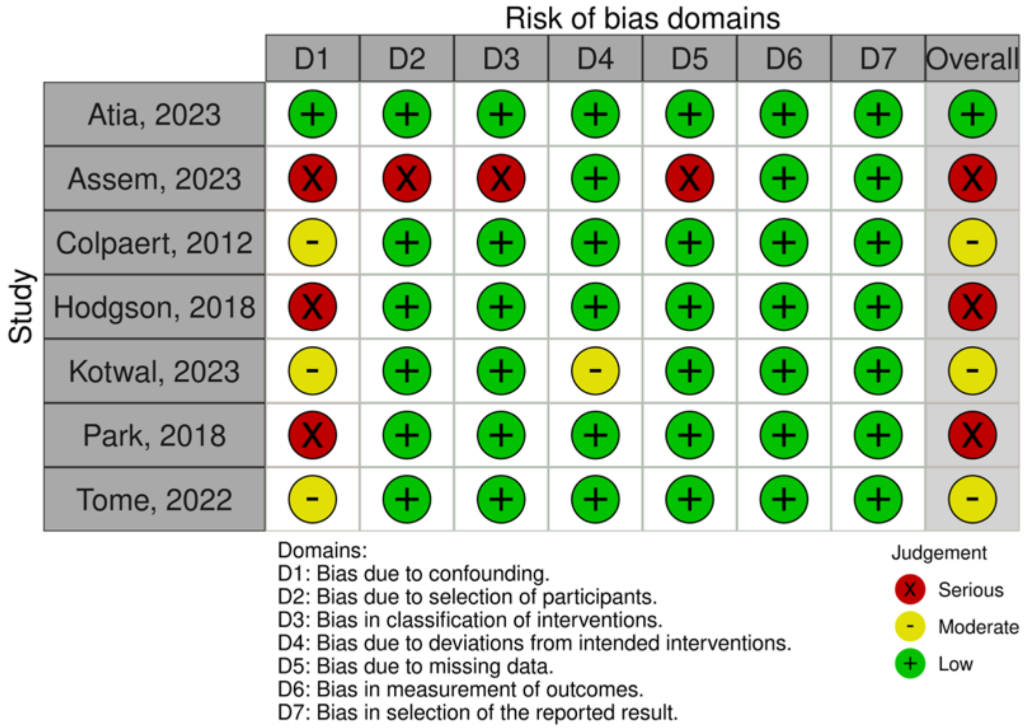

© 2024 Chen JJ et al. *JAMA Network Open*.

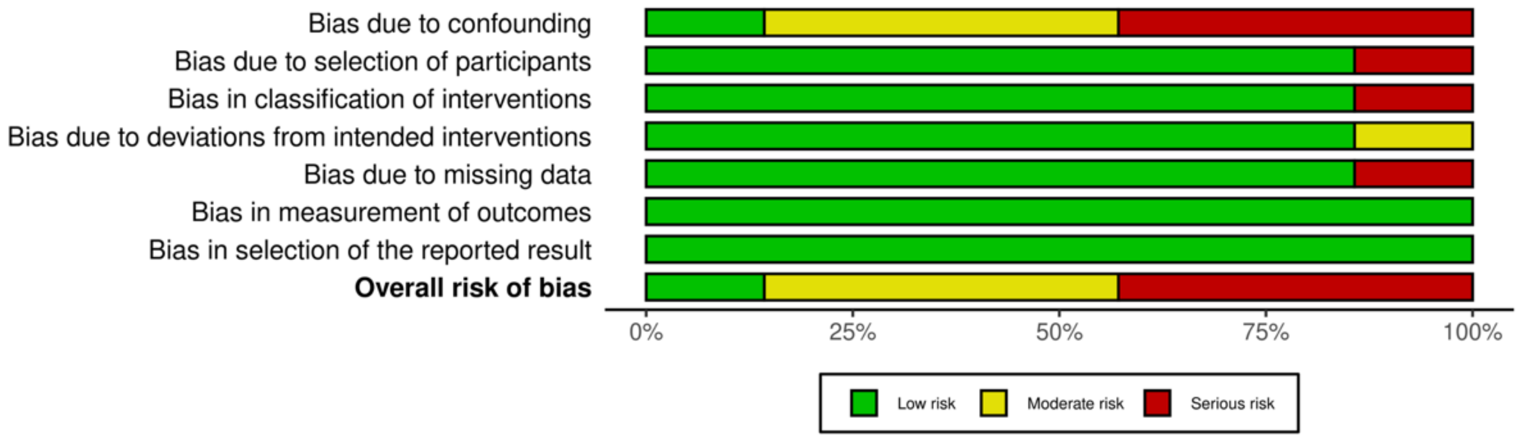

© 2024 Chen JJ et al. *JAMA Network Open*.

eFigure 4. Forest Plot Illustrating the Association of Acute Kidney Injury (AKI) Electronic Alerts (e-Alerts) With Dialysis and Kidney Recovery after AKI

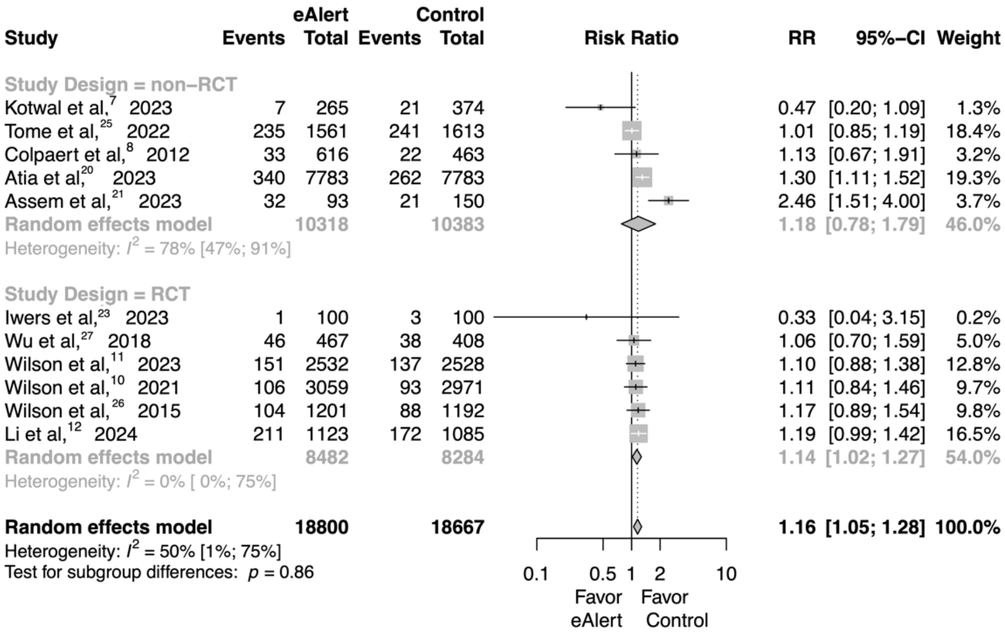

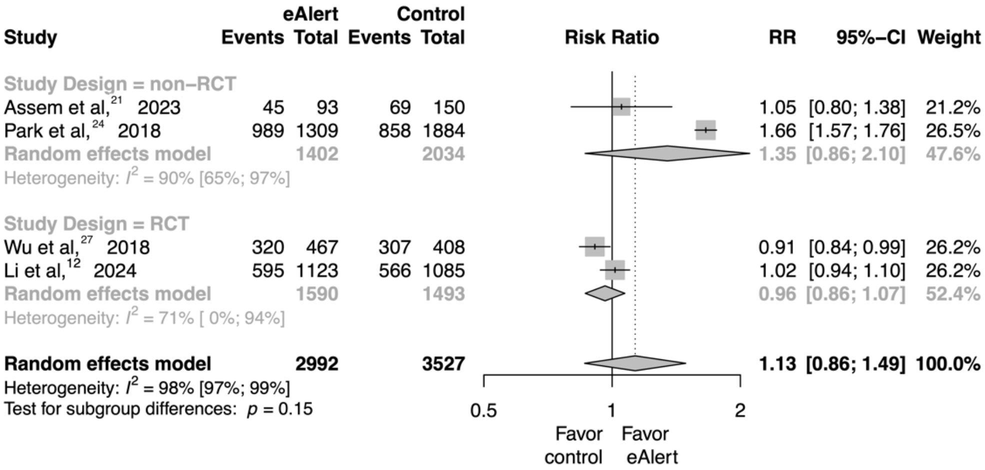

© 2024 Chen JJ et al. *JAMA Network Open*.

eFigure 5. Forest Plot Illustrating the Association of Acute Kidney Injury (AKI) Electronic Alerts (e-Alerts) With ACEI/ARB Prescription and Fluid Prescription After AKI

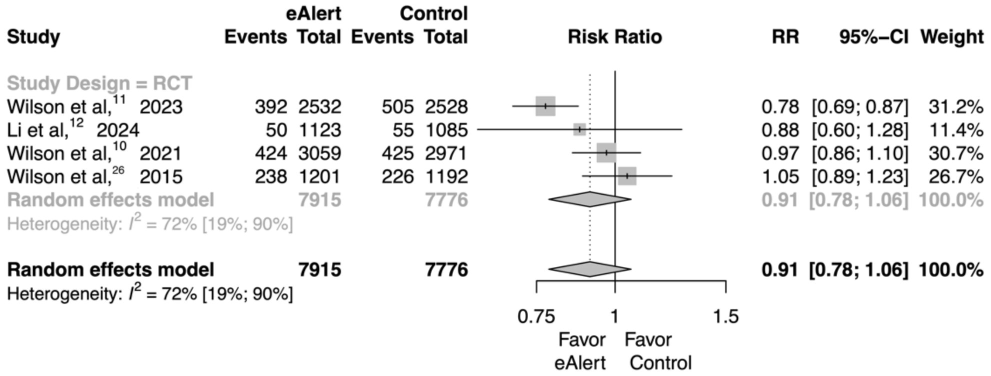

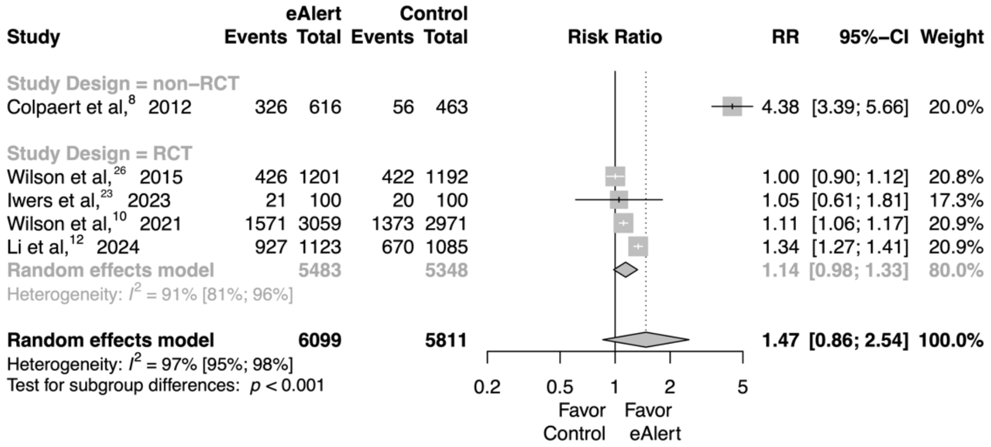

© 2024 Chen JJ et al. *JAMA Network Open*.

eFigure 6. Subgroup Analysis for AKI Progression

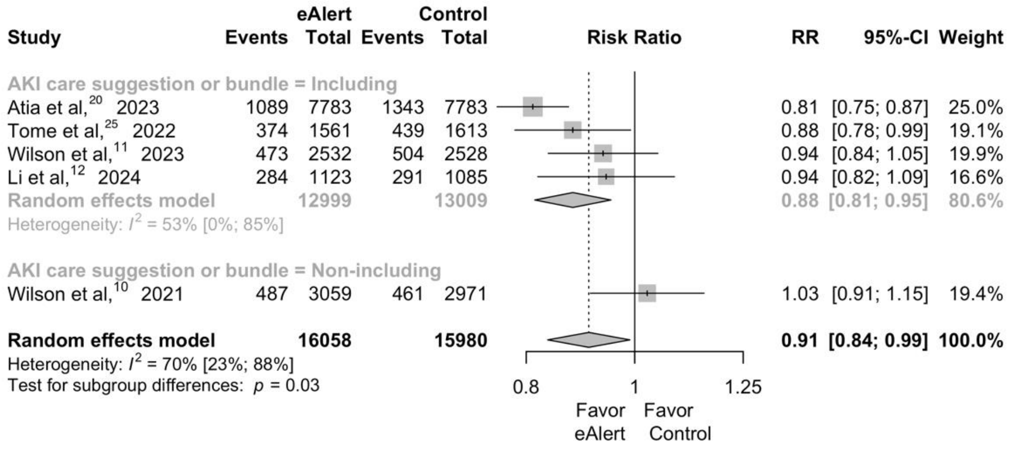

eFigure 7. Subgroup Analysis for Mortality

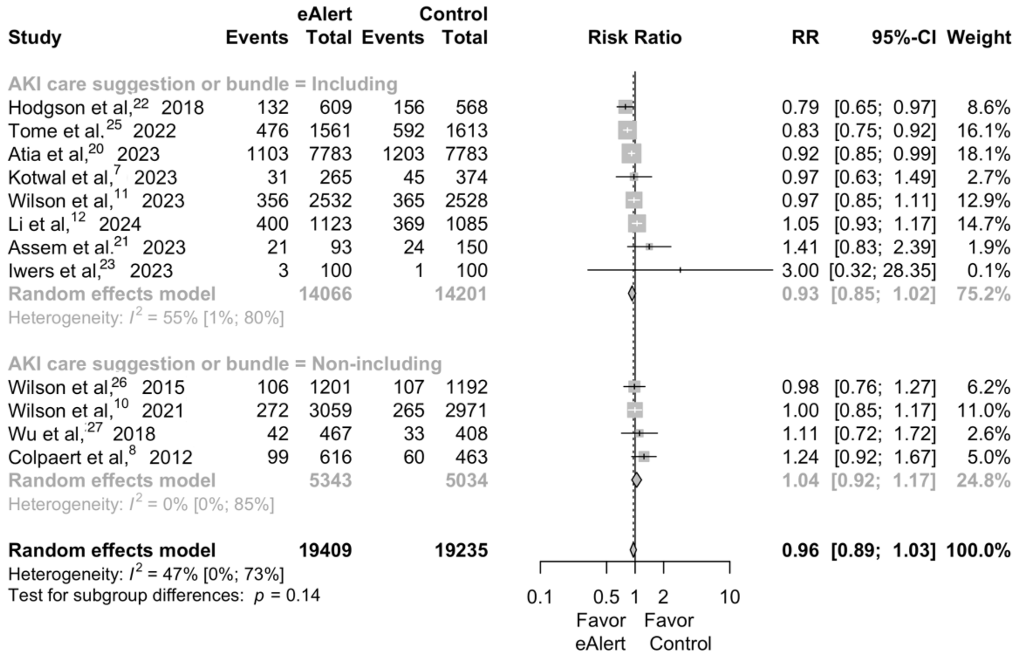

eFigure 8. Subgroup Analysis for Dialysis

© 2024 Chen JJ et al. *JAMA Network Open*.

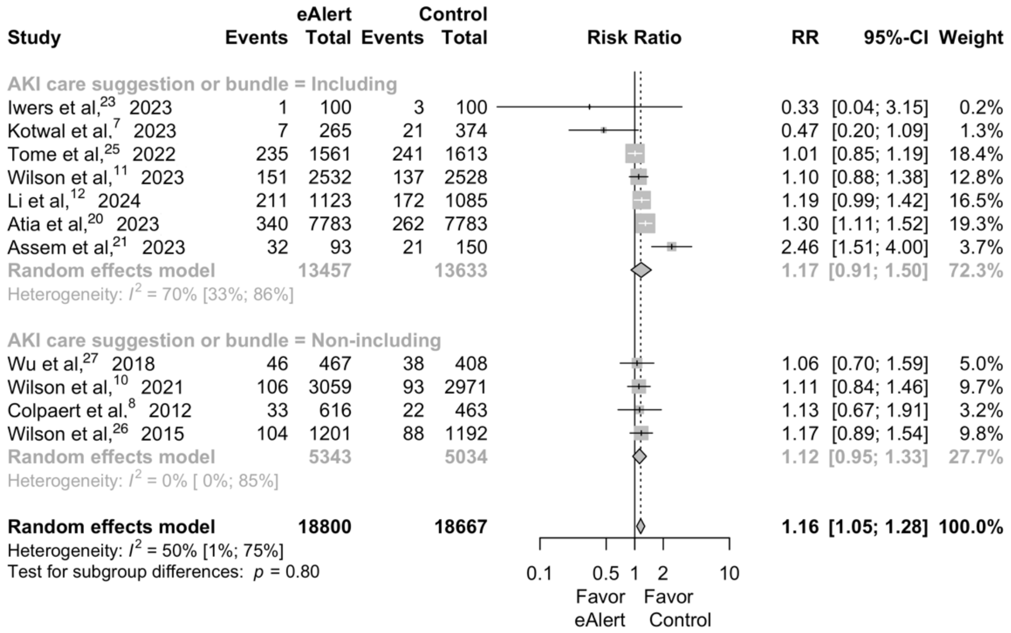

eFigure 9. Subgroup Analysis for Kidney Recovery

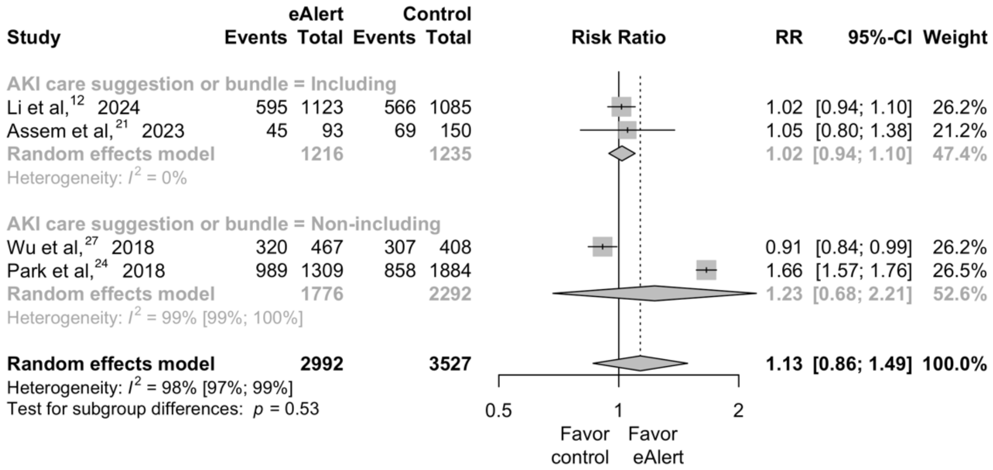

eFigure 10. Subgroup Analysis for Nephrologist Consultation

© 2024 Chen JJ et al. *JAMA Network Open*.

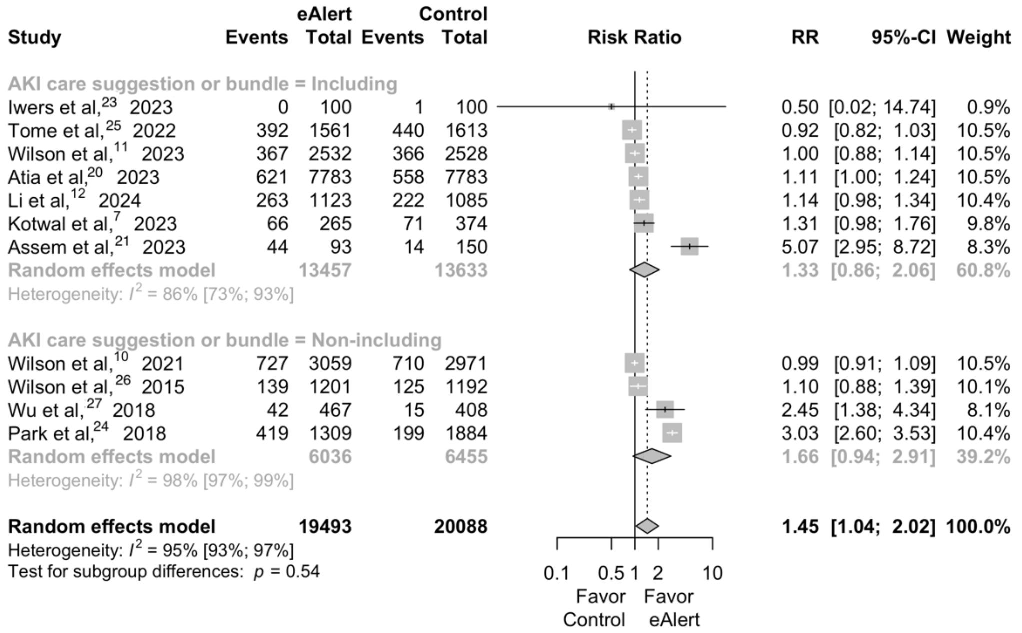

eFigure 11. Subgroup Analysis for NSAID Exposure After AKI

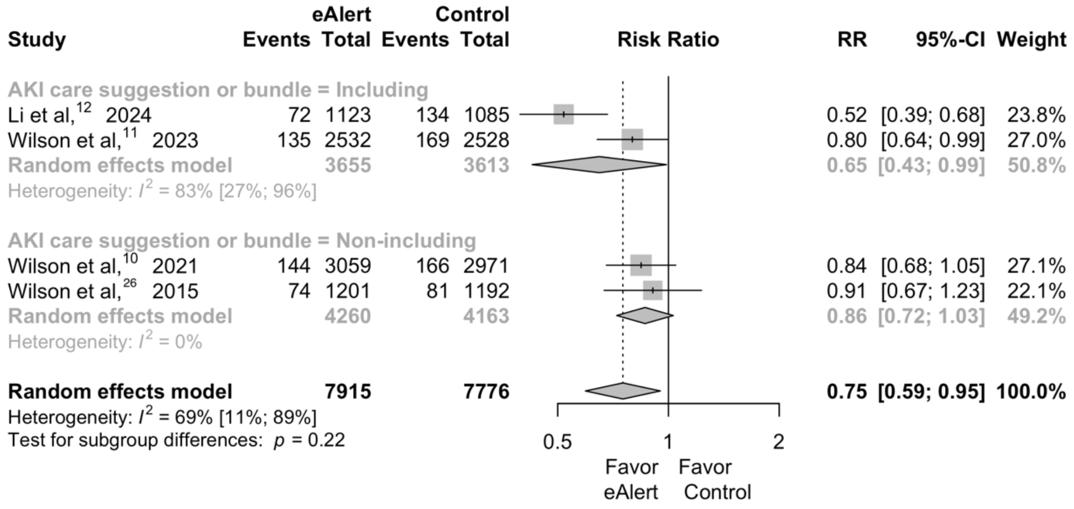

eFigure 12. Subgroup Analysis for Hospital Length of Stay

© 2024 Chen JJ et al. *JAMA Network Open*.

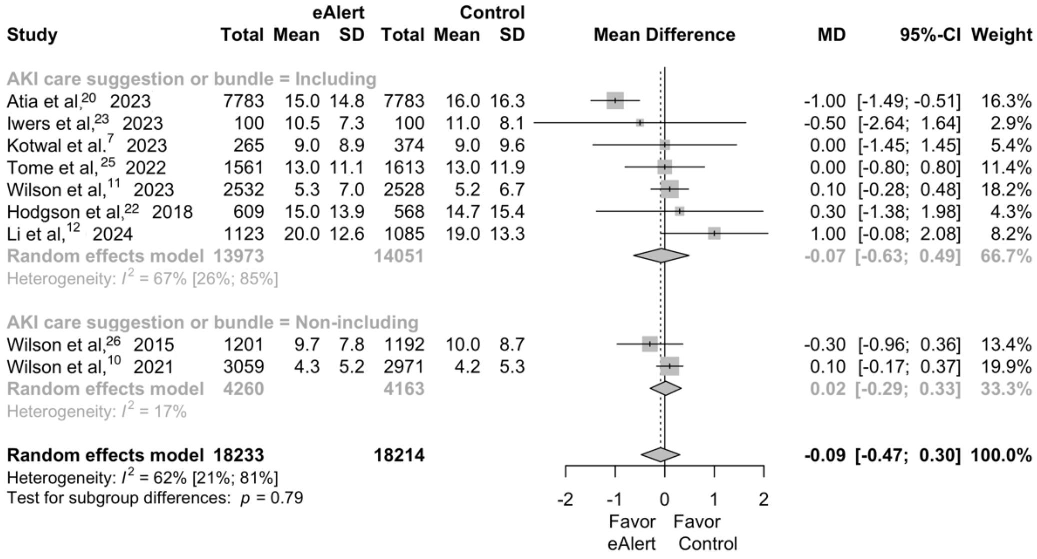

eFigure 13. Subgroup Analysis for Medical Costs

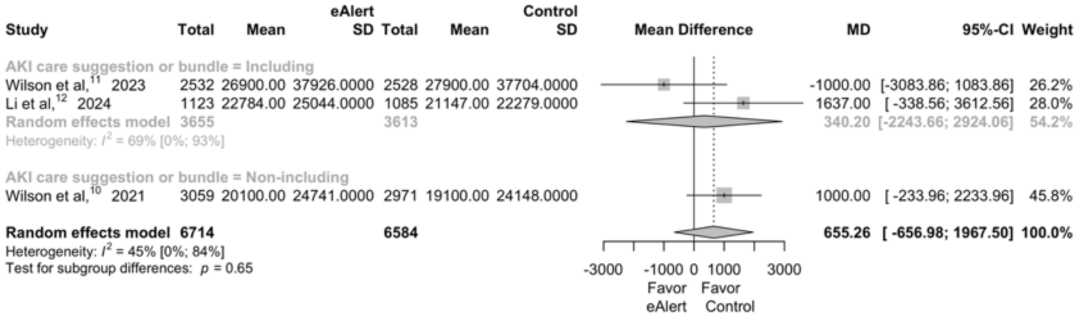

eFigure 14. Subgroup Analysis for AKI Documentation

© 2024 Chen JJ et al. *JAMA Network Open*.

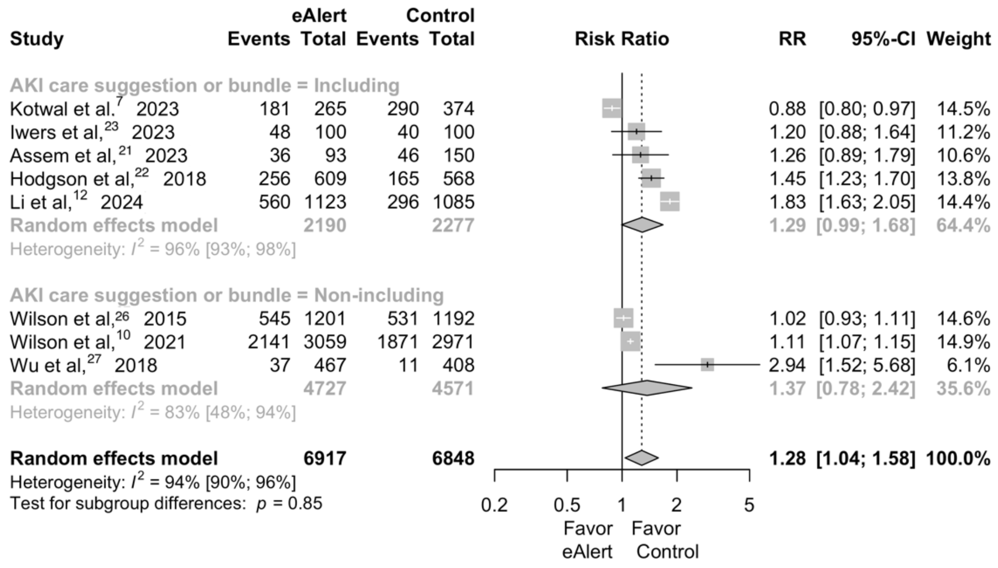

eFigure 15. Subgroup Analysis for Fluid prescription

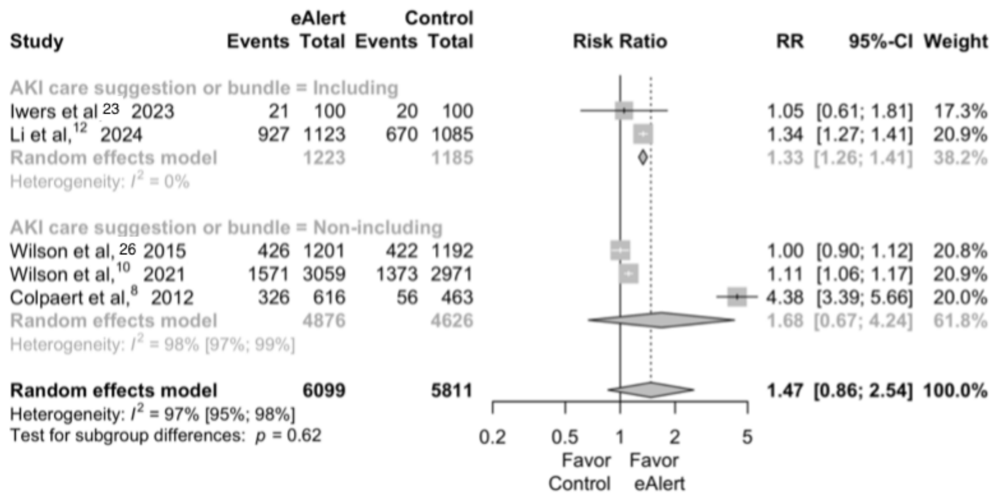

© 2024 Chen JJ et al. *JAMA Network Open*.

eFigure 16. Subgroup Analysis for ACEI/ARB Exposure

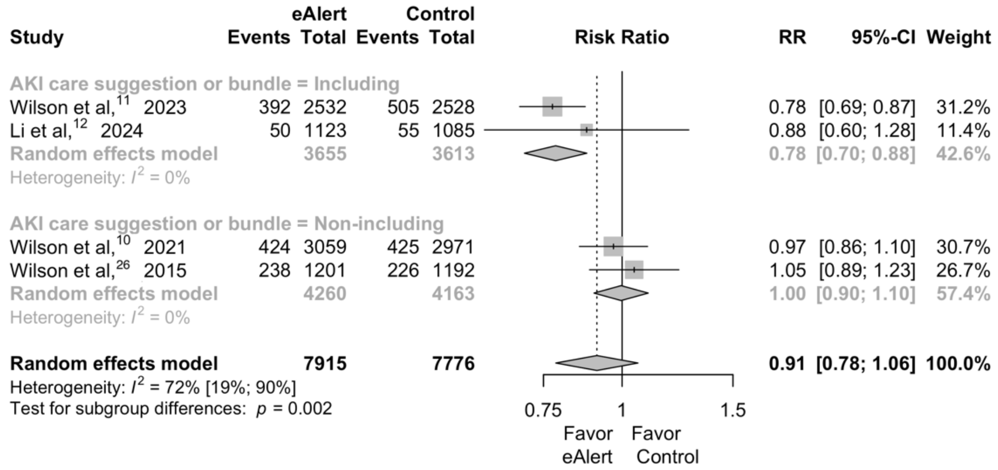

eFigure 17. Subgroup Analysis for Mortality With Different Follow-Up Period

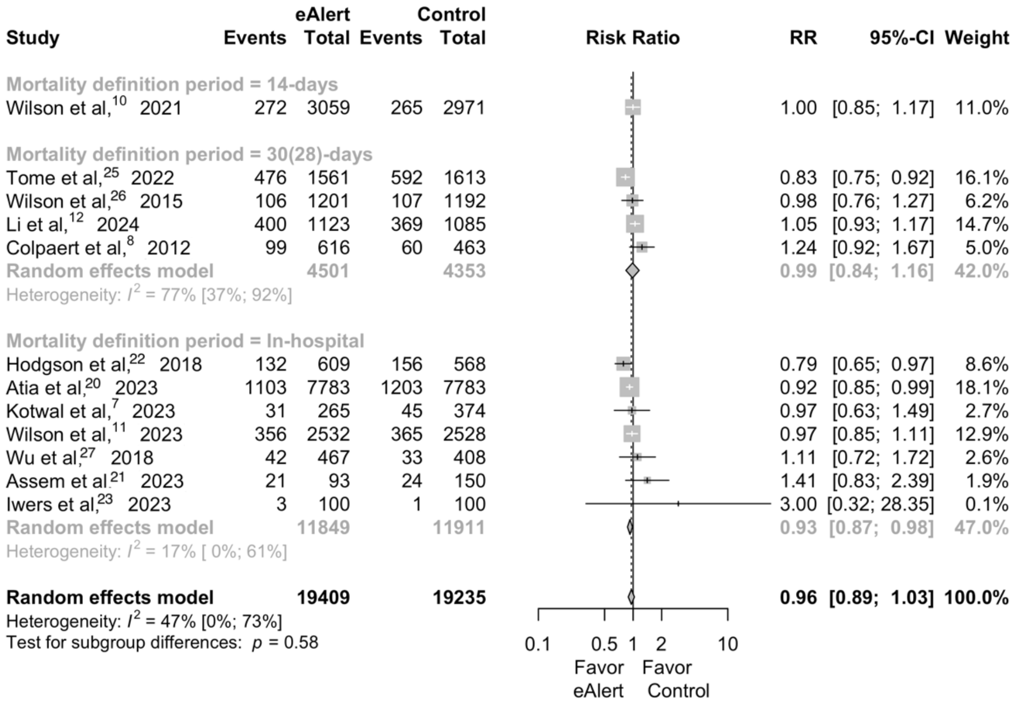

eFigure 18. Trial Sequential Analysis for Mortality

© 2024 Chen JJ et al. *JAMA Network Open*.

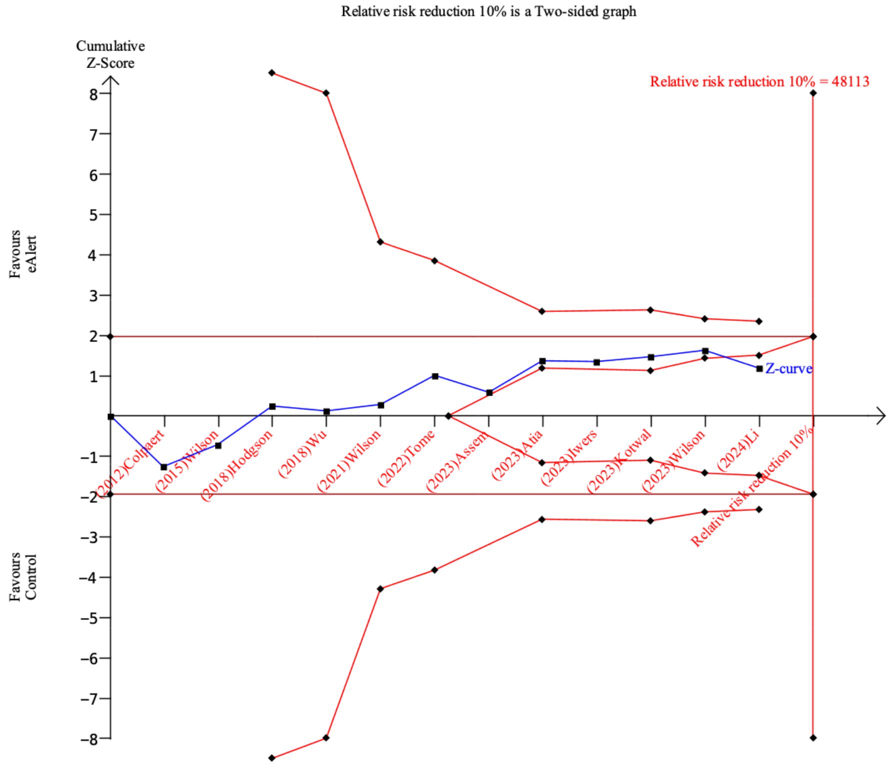

eFigure 19. Trial Sequential Analysis for AKI Progression

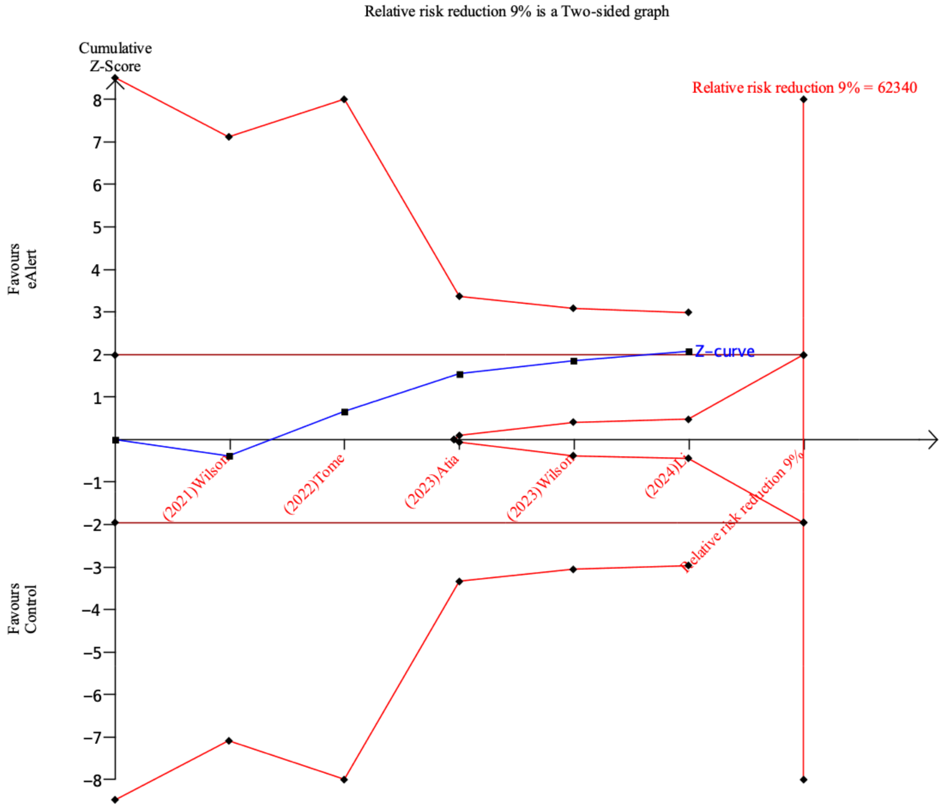

© 2024 Chen JJ et al. *JAMA Network Open*.

eFigure 20. Trial Sequential Analysis for Dialysis

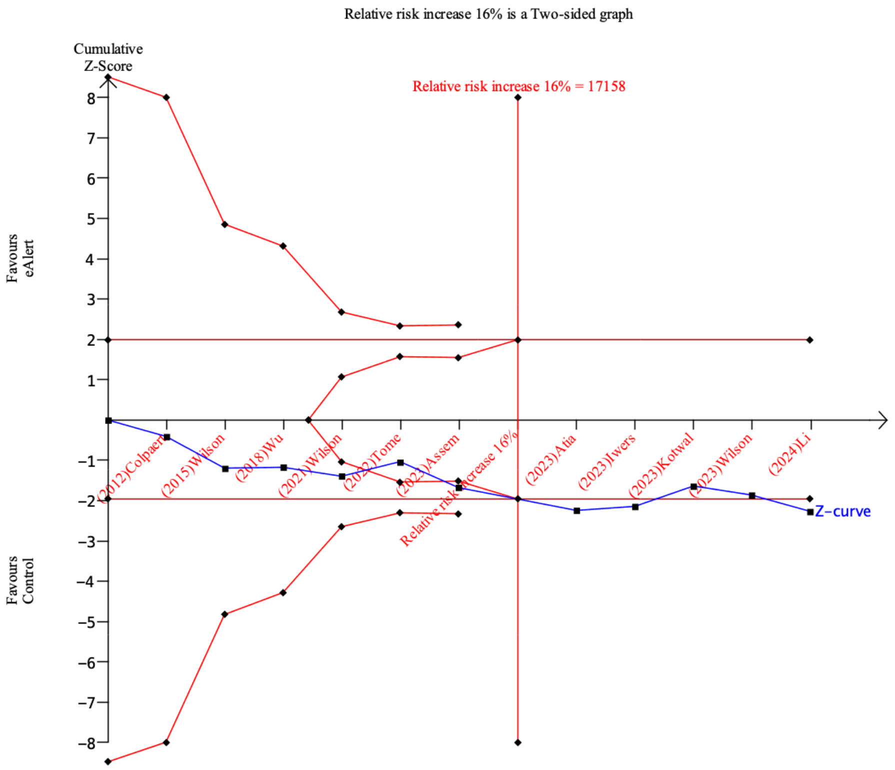

eFigure 21. Trial Sequential Analysis for Kidney Recovery

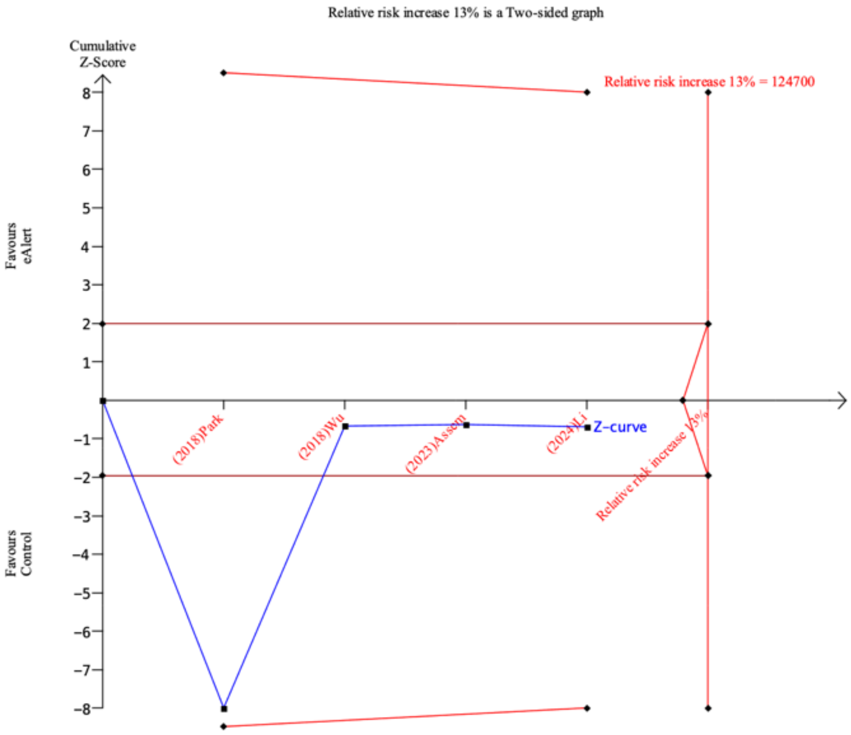

© 2024 Chen JJ et al. *JAMA Network Open*.

eFigure 22. Trial Sequential Analysis for Nephrologist Consultation

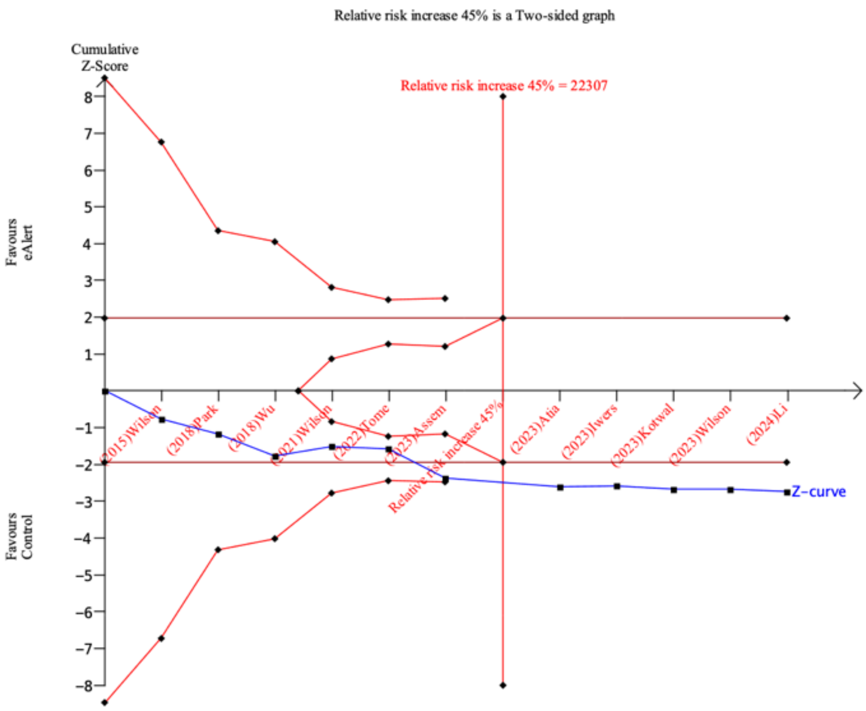

eFigure 23. Trial Sequential Analysis for NSAID Exposure After AKI

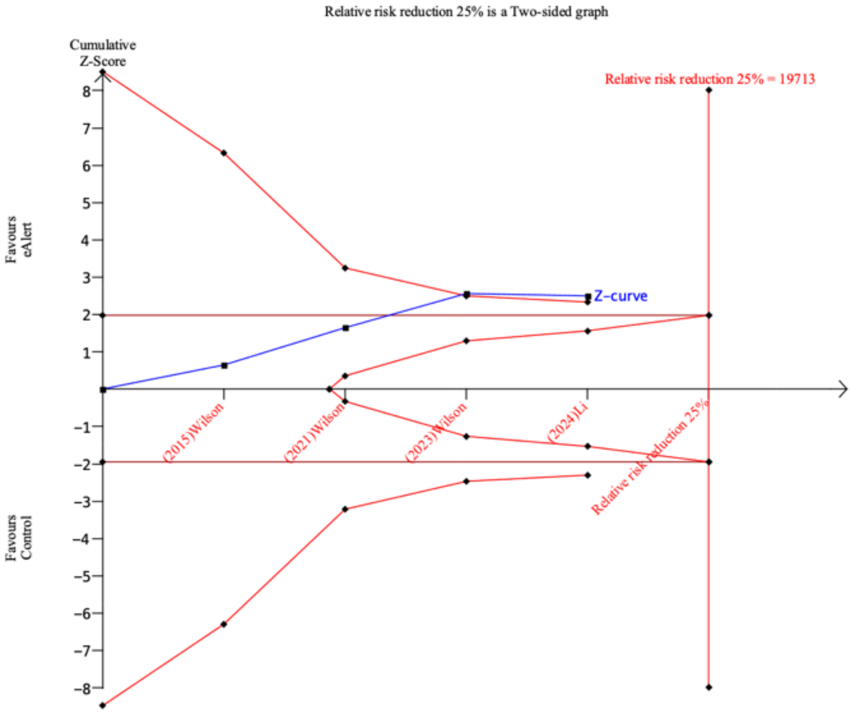

© 2024 Chen JJ et al. *JAMA Network Open*.

eFigure 24. Trial Sequential Analysis for AKI Documentation

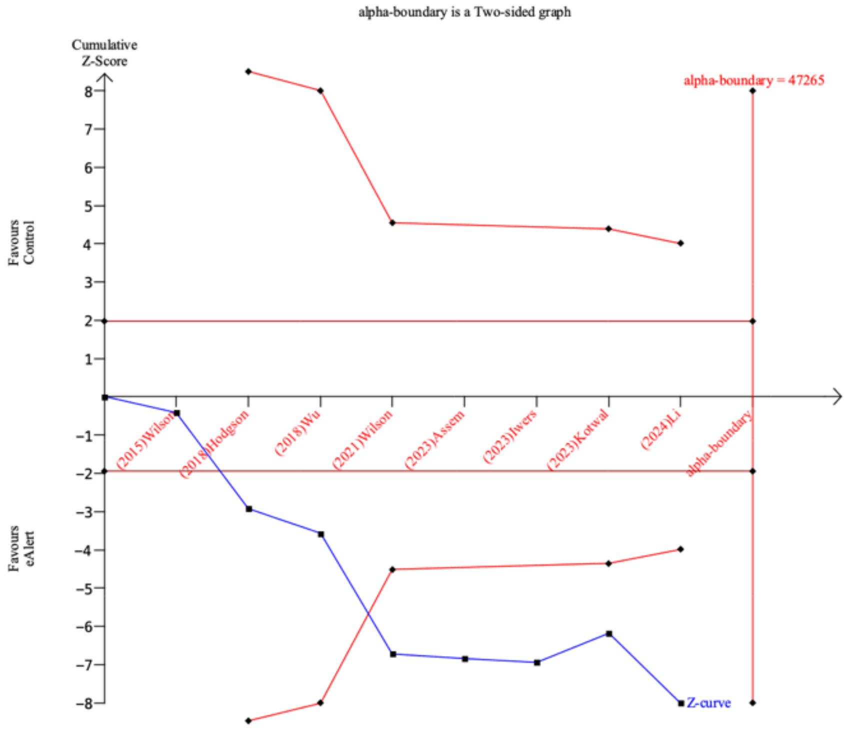

© 2024 Chen JJ et al. *JAMA Network Open*.

eFigure 25. Funnel Plots

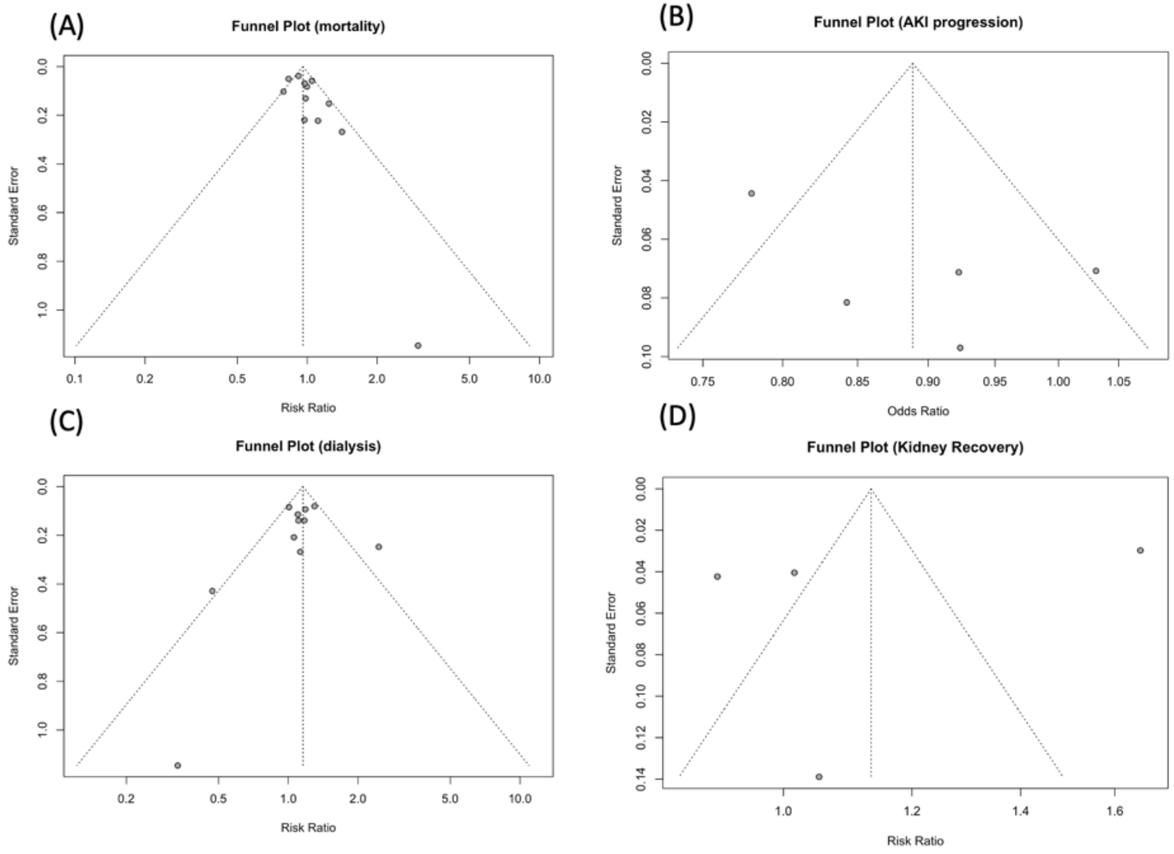

© 2024 Chen JJ et al. *JAMA Network Open*.

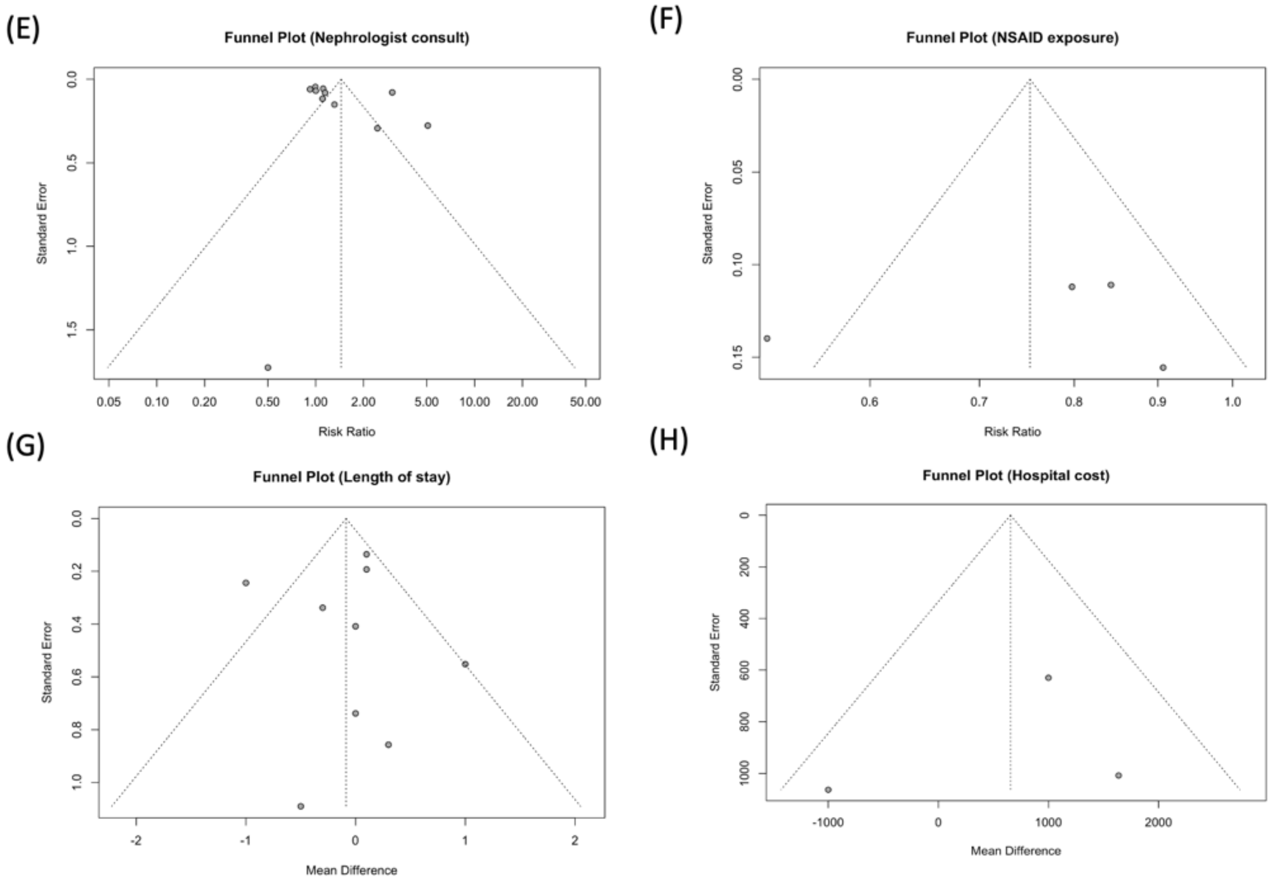

© 2024 Chen JJ et al. *JAMA Network Open*.

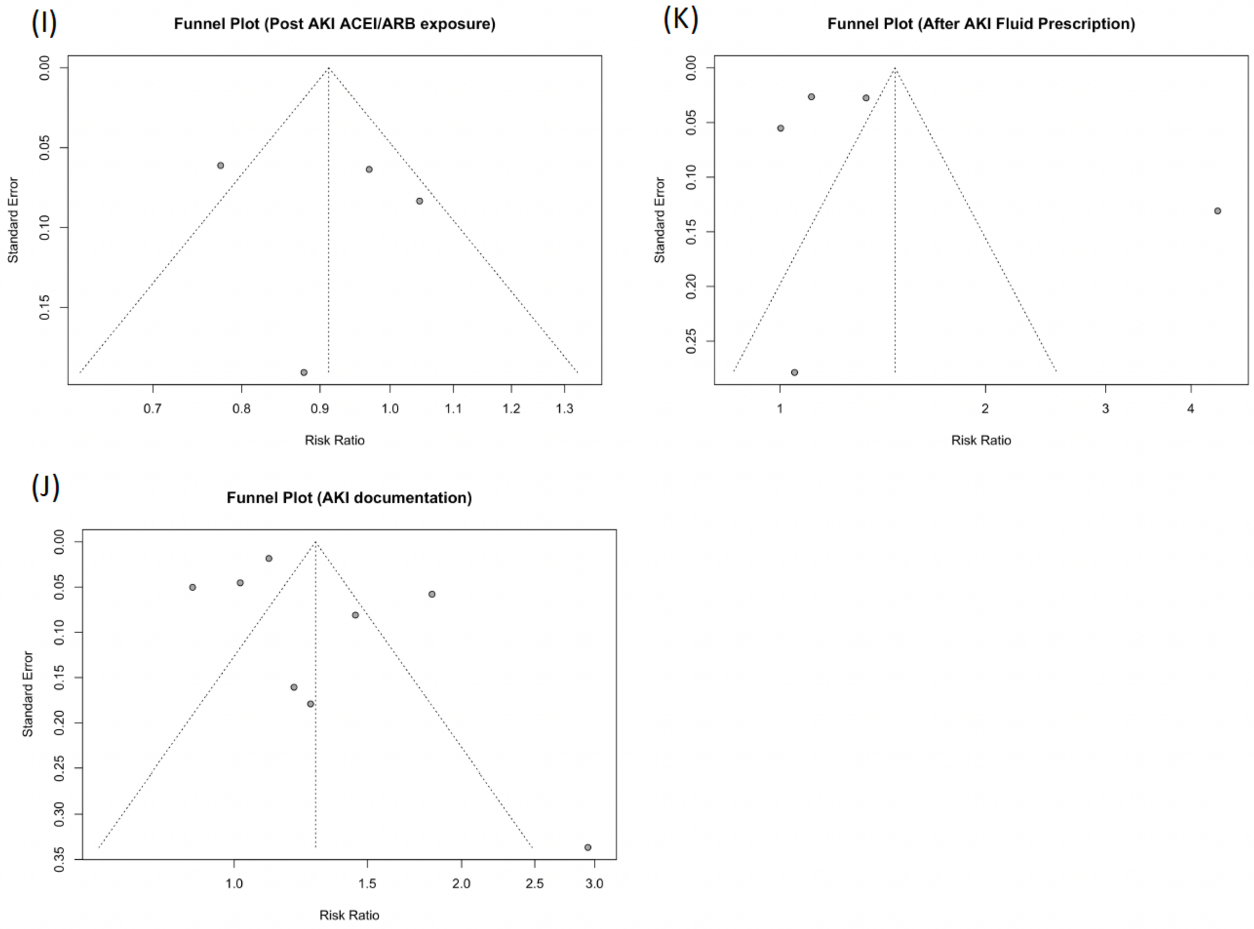

© 2024 Chen JJ et al. *JAMA Network Open*.

Mortality (A), AKI progression (B), Dialysis (C), Kidney Recovery (D), Nephrologist Consultation (E), NSAID Exposure after AKI (F), Hospital Length of Stay (G), Medical Costs (H), ACEI/ARB Exposure after AKI (I), AKI Documentation (J), Fluid Prescription after AKI (K)

© 2024 Chen JJ et al. *JAMA Network Open*.

eAppendix 1. Supplemental Method for Trial Sequential Analysis

We set a type I error rate at 5% and statistical power at 80%, employing a two-sided boundary with a penalty of 2. For the four primary outcomes, we established risk reduction ratios (effect sizes) of 10% for mortality, 9% for AKI progression, 16% for dialysis, and 13% for AKI recovery. For three secondary outcomes (nephrologist consultation, NSAID exposure post-AKI, AKI documentation) that showed significant effects in the conventional meta-analysis, TSA was performed with expected risk reduction ratios of 45%, 25% and increased reduction ratios of 28%, respectively. A random-effects model using the Biggerstaff–Tweedie (BT) method was applied, with heterogeneity for TSA derived from this model.

eAppendix 2. Quality of Included Studies

For RCTs, RoB 2 identified two studies (Iwers, 2023; Wu, 2018)[24,28] with high or some concerns regarding randomization in domain 1. Wu's study[28] also raised concerns about the selection of reported results, specifically the post-discharge follow-up analysis as per trial registration. No study exhibited other biases. The overall RCT quality was ranked as low risk for 4 studies (66.7%), some concern for 1 study (16.7%), and high concern for 1 study (16.7%) (**eFigure 2A & 2B**).

For non-RCTs, one study (Atia, 2023)[21] showed low concern for bias due to confounding in domain 1. Three studies (Assem, 2023; Hodgson, 2018; Park, 2018)[22,23,25] had serious concerns due to uncontrolled or unmeasured significant confounding. Three others (Colpaert, 2012; Kotwal, 2023; Tome, 2022) [7,8,26] had moderate concerns in domain 1 for not adequately controlling for significant confounding. Assem et al.'s study[22] also had serious concerns regarding participant selection (domain 2), intervention classification (domain 3), and missing data (domain 5), with only 77.8% considered accurate AKI e-Alerts reporting the outcome of interest. Thus, the overall quality of non-RCTs was low risk for 1 study (14.3%), moderate concern for 3 studies (42.9%), and high concern for 3 studies (42.9%) (**eFigure 3A & 3B**).

© 2024 Chen JJ et al. *JAMA Network Open*.

eDocument 3. Certainty of Evidence Assessment for AKI Progression

Only one of the five enrolled trials evaluating AKI progression had a moderate risk of bias, while 80% of the trials had a low risk of bias. Therefore, we assessed the overall risk of bias as not serious. Although the I^2 was relatively high, two prespecified subgroup analyses (design: RCT vs. non-RCT; trials with care bundle vs. trials without care bundle) demonstrated significant subgroup differences. As a result, we also assessed the inconsistency domain as not serious.

© 2024 Chen JJ et al. *JAMA Network Open*.
