# Supplementary material for: Electronic Alert Systems for Patients With Acute Kidney Injury: A Systematic Review and Meta-Analysis
Source: JAMA Netw Open. 2024 Aug 27;7(8):e2430401. doi: 10.1001/jamanetworkopen.2024.30401 (PMC11350470; doi:10.1001/jamanetworkopen.2024.30401)
Supplement: Supplement 2. — Data Sharing Statement [file jamanetwopen-e2430401-s002.pdf]

## Data Sharing Statement

Chen. Electronic Alert Systems for Patients With Acute Kidney Injury. *JAMA Netw Open*. Published August 27, 2024. doi:10.1001/jamanetworkopen.2024.30401

**Data**
**Data available:** No
